# Quantitative ^23^Na magnetic resonance imaging in the abdomen at 3 T

**DOI:** 10.1007/s10334-024-01167-6

**Published:** 2024-06-01

**Authors:** Jonathan Richard Birchall, Ines Horvat-Menih, Joshua Daniel Kaggie, Frank Riemer, Arnold Julian Vinoj Benjamin, Martin John Graves, Ian Wilkinson, Ferdia Aidan Gallagher, Mary Anne McLean

**Affiliations:** 1 Department of Radiology, University of Cambridge, Cambridge, UK; 2 Department of Radiology, Mohn Medical Imaging and Visualization Centre, Haukeland University Hospital Helse Bergen, Bergen, Norway; 3 Cambridge Cardiovascular, University of Cambridge, Cambridge, UK

**Keywords:** Sodium MRI, Quantification, Tissue sodium content, Relaxation, Abdomen

## Abstract

**Objectives:**

To assess the feasibility of sodium-23 MRI for performing quantitative and non-invasive measurements of total sodium concentration (TSC) and relaxation in a variety of abdominal organs.

**Materials and methods:**

Proton and sodium imaging of the abdomen was performed in 19 healthy volunteers using a 3D cones sequence and a sodium-tuned 4-rung transmit/receive body coil on a clinical 3 T system. The effects of *B*_1_ non-uniformity on TSC measurements were corrected using the double-angle method. The long-component of ^23^Na *T*_2_* relaxation time was measured using a series of variable echo-times.

**Results:**

The mean and standard deviation of TSC and long-component ^23^Na *T*_2_* values were calculated across the healthy volunteer group in the kidneys, cerebrospinal fluid (CSF), liver, gallbladder, spleen, aorta, and inferior vena cava.

**Discussion:**

Mean TSC values in the kidneys, liver, and spleen were similar to those reported using ^23^Na-MRI previously in the literature. Measurements in the CSF and gallbladder were lower, potentially due to the reduced spatial resolution achievable in a clinically acceptable scan time. Mean long-component ^23^Na *T*_2_* values were consistent with previous reports from the kidneys and CSF. Intra-population standard error was larger in smaller, fluid-filled structures due to fluid motion and partial volume effects.

**Supplementary Information:**

The online version contains supplementary material available at 10.1007/s10334-024-01167-6.

## Introduction

Magnetic resonance imaging (MRI) is a powerful tool for routine and exploratory clinical diagnostic imaging of the human body without the need for ionising radiation or invasive biopsies. Whilst routine proton MRI techniques are widely applicable in the imaging of both tissue structure and function owing to the abundance of protons in body fat and water, imaging of other biologically relevant and MR-active X-nuclei such as sodium-23 is also possible. Existing techniques for quantification of sodium content such as flame photometry [1, 2], ion-specific electrochemical potentiometric techniques [3, 4], or inductively coupled plasma optical emission spectroscopy (ICP–OES)[5] are well-documented in the intracellular and extracellular compartments [6, 7], but all require tissue acquisition which is invasive. Non-invasive techniques such as urinary osmolarity have been used to study the corticomedullary gradient (CMG) in the kidneys, but these provide only indirect and non-specific information on kidney function, and are not widely translatable to other organs [8].

In addition to hydrolysis of ATP and the phosphorylation of the Na^+^/K^+^ pump, endogenous sodium exchange between the intracellular and the extracellular compartments plays an essential role in numerous biological pathways, including regulation of blood volume, pressure and pH, as well as neuronal activation and maintenance of the cell resting potential [9]. However, excessive dietary sodium intake has been linked to numerous health risks, with increased blood pressure in particular being a contributing factor in the prevalence of heart disease, chronic kidney disease and stroke among others [10–12]. Previous histological studies have shown the total sodium concentration (TSC) to be elevated by more than 50% in malignant brain and breast tumours in humans in comparison with healthy tissue, and changes in sodium content are thought to correlate with successful treatment [13, 14]. In addition, there is a clinical need for the differentiation of tumour types and cellular sodium uptake and retention may provide an opportunity to discriminate more aggressive from less aggressive tumours.

Quantitative analysis of the combined sodium content from both the intracellular and extracellular compartments using MRI has been demonstrated in a number of prior studies [9, 15–18], and further progress in the field is arising from advancements in dedicated ^23^Na-tuned radiofrequency (RF) coil design, pulse sequence development and data analysis [18–28]. There is, therefore, an interest in establishing ^23^Na-MRI as a quantitative imaging biomarker for cellular integrity and metabolism in disease diagnosis. Recent human imaging studies have included the determination of tumour cellularity in ovarian cancer [29], quantification of sodium in prostate tumours [30], linking of elevated skin sodium content to multiple sclerosis [31] and psoriasis [32], as well as the measurement of tissue sodium accumulation in chronic kidney disease (CKD) [33, 34]. Ex vivo ^23^Na MR spectroscopy of human tissue has also been utilised to observe a reduction in skin sodium content in Type 2 diabetes [35], and determination of the salt-sensitivity of blood pressure (SSBP) using ^23^Na MRI may be potentially useful as a non-invasive, quantitative imaging biomarker for hypertension [36, 37].

In addition, measurements of the transverse relaxation time (*T*_2_*) could potentially provide information on tissue oxygenation; measuring the inverse relaxation rate (*R*_2_* = 1/*T*_2_*) in proton MR has shown elevation in the presence of paramagnetic deoxyhaemoglobin [38]. Quantitative proton *T*_2_* mapping has been demonstrated as a potential biomarker in the imaging of tumour hypoxia [39, 40] and in the prognostic staging of rectal cancer [41]. Quantification of ^23^Na *T*_2_* in humans has proven challenging due to additional relaxation effects arising from the quadrupolar nature of the ^23^Na nucleus (*I *= 3/2), where spin relaxation follows a bi-exponential trend with one decay component being substantially longer than the other [49, 50]. Multiple quantum filtering techniques for separating these decay components have been demonstrated in the human brain, but have yet to find wider application due to the order of magnitude lower signal intensity relative to the single quantum spin-density-weighted imaging [51, 52]. In addition, the larger and less-efficient coils employed in abdominal imaging necessitate longer pulse widths to achieve desired flip angles, by which point the short *T*_2_* relaxation component has already decayed to a significant extent, limiting the achievable SNR. To this end, in vivo evaluation of *T*_2_* estimation in other organs and fluid-filled structures for the first time is of interest to complement previous work in the healthy human kidneys [42, 43].

In this study, we report estimates of total ^23^Na content (TSC) and long-component transverse relaxation (*T*_2_*) in eight organs and fluid-filled structures within the abdomen of healthy volunteers (*N* = 19, 12 M/7F).

## Materials and methods

This study was approved by the local ethical review committee (reference number 08-H0311-117).

### Data acquisition

**Fig. 1:**
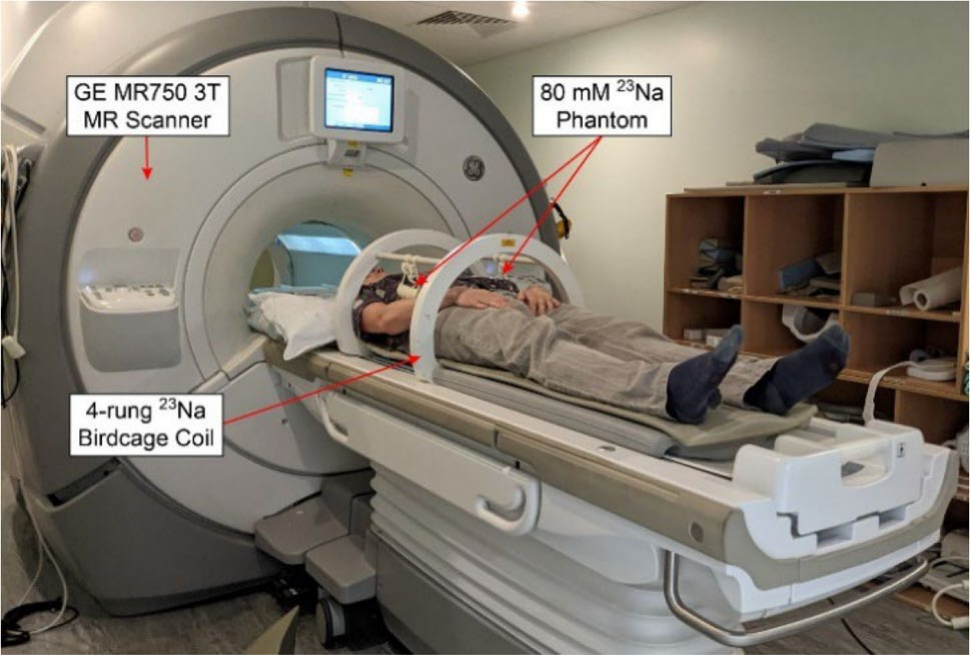
Orientation of healthy volunteers, the ^23^Na birdcage T/R body coil and 80 mM agar gel phantoms within the 3 T body MRI scanner

Axial ^23^Na MR images of the abdomen were acquired using a novel birdcage transmit/receive body coil, possessing a large (~48 cm) field-of-view (FOV) which facilitated uniform excitation over a significant portion of the abdomen in subjects with a range of body habitus, as we have previously described [44]. Corresponding axial proton images were acquired at 3 T using a clinical GE MR750 system (GE Healthcare, Waukesha WI). A pair of 50 mL Falcon tubes, each containing an 80 mM agar gel sodium chloride phantom, were suspended from the upper rungs on either side of the ^23^Na birdcage coil (Fig. 1) to serve as a calibration standard for signal intensity. In later subjects, two further identical phantoms were placed underneath the patient to the left and right sides of the bed centre, to serve as an additional point of reference.

Healthy volunteers were situated headfirst and supine inside the ^23^Na birdcage coil, with the centre of the coil positioned over the kidneys, as confirmed using a fast gradient echo (FGRE) ^1^H localiser sequence. Anatomical fat/water ^1^H imaging was achieved using an axial breath-hold 3D T_1_-weighted sequence (LAVA-Flex GRE) with the MR system body coil and 32 locations each (TEs = 1.1/2.2 ms, TR = 3.7 ms, flip = 15°, averages = 1, matrix = 256 × 192, phase FOV = 0.7, FOV = 40 cm). Shims were optimized over a large (~16 cm diameter) cuboid region and these shim settings were retained in the following multinuclear scans.

A multinuclear Bloch–Siegert-based calibration scan sequence [45] was prescribed in the centre of the coil to determine the centre frequency of ^23^Na and suitable transmit and receive gain values; all values were kept constant for the duration of the scanning session to ensure consistent intensity scaling. A pair of low-resolution images were acquired using a 3D cones trajectory (FOV = 48 cm, TR = 150 ms, TE = 0.705 ms, flip angle = 40/80°, trajectory resolution = 9 × 9 × 9 mm, 197 transients, averages = 3, 125 kHz full receiver bandwidth, total duration = 1:28 min per sequence) to perform *B*_1_ non-uniformity correction using the double-angle method (DAM) [46–48].

A higher-resolution 3D conical trajectory (FOV = 48 cm, TR = 100 ms, TE = 0.705 ms, flip angle = 70°, voxel size = 4 × 4 × 8 mm, 1402 transients, averages = 5, 167 kHz full receiver bandwidth, total duration = 11:41 min) was used to acquire maps of ^23^Na signal intensity across the abdomen [49]. Finally, a series of six low-resolution series were acquired with a further 3D cones trajectory (FOV = 48 cm, TR = 100 ms, flip angle = 70°, trajectory resolution = 9 × 9 × 9 mm, 197 transients, averages = 4, 125 kHz full receiver bandwidth, total duration = 1:19 min per series) were performed at a range of echo times (TEs = 0.705, 1.5, 2, 4, 8 and 16 ms) to generate quantitative maps of the ^23^Na *T*_2_* relaxation time constant across the abdomen. Low-resolution images spatially matched the location and FOV of the high-resolution counterpart in each subject.

## MATLAB data analysis

All data analysis and region drawing were performed in MATLAB (Mathworks, Natick MA).

### *B*_1_ field inhomogeneity correction

For each subject, a map of the relative *B*_1_ value at each voxel was generated from the corresponding intensity ratio of high (80°)-to-low (40°) flip-angle intensity in the two low-resolution DAM series data sets as

1 $${\text{rel}}B_1 \left( {x,y,z} \right) = {\text{cos}}^{ - 1} {{\left( {\frac{{I\left( {x,y,z} \right)_{{\text{high}}} }}{{2~ \cdot ~I\left( {x,y,z} \right)_{{\text{low}}} }}} \right)} / {\alpha _{{\text{low}}} }}$$

where *I*_high_ and *I*_low_ are the signal intensities at each voxel in the high and low flip-angle data sets, respectively, and *α*_low_ is the lower of the two flip-angles used (nominally 40° in our protocol). A 3D median filter (kernel size = 5 × 5 × 5 voxels) was applied to maximise the signal-to-noise ratio (SNR) whilst preserving edge information. Example output from the 3D median filtering process in MATLAB is presented in Figure S1 of the Supporting Information (SI). Thresholding was performed, so that the signal intensity ratio was made equal to zero for voxels in the high flip-angle data set, possessing an intensity less than 10% of the maximum observed value. Finally, values were normalised to generate a value of 1000 when the actual and nominal flip angles were equal.

It should be noted that for some of the healthy volunteers, *B*_1_ mapping data were not acquired as part of the imaging protocol. To perform *B*_1_ non-uniformity correction in these subjects, a population average estimate *B*_1_ map was created by taking the median non-zero *B*_1_ value at every voxel across all the remaining subjects (*N* = 17) and using this as a reference when determining the effective total sodium concentration.

Three-dimensional maps of the *B*_1_-corrected total tissue sodium concentration (TSC) were calculated from the high-resolution 3D cones trajectory series in each of the *N* = 19 healthy volunteers as 80 mM multiplied by the ratio of the pixel intensity to the average intensity in a region of interest (ROI) drawn inside the calibration phantom. A coronal maximum intensity projection (MIP), shown in Figure S2 of the Supporting Information, was additionally acquired in one subject, but was not used for the purposes of TSC estimation.

However, due to non-linear image distortions which differed between the DAM and high-resolution ^23^Na series (shown in Figure S3 of the Supporting Information and discussed in more detail in SI Section 2), a single image transform could not be applied to adequately register both volumes to the ^1^H anatomical series. In addition, the tissue contrast of the DAM series was insufficient to reliably register specific regions and structures to the corresponding ^1^H anatomical image. Generation of *B*_1_-corrected TSC maps across the whole abdomen was, therefore, considered unreliable. To this end, TSC calculation was instead performed by undertaking *B*_1_ non-uniformity correction on a per-region basis, drawing a region for each organ or fluid-filled structure in both the high-resolution ^23^Na image and the low-resolution DAM *B*_1_ map. Region TSC was then calculated by accounting for the receive and transmit *B*_1_ profiles as follows:

2 $$C_{{\text{organ}}} = C_{{\text{ref}}} \cdot \frac{{I_{{\text{organ}}} }}{{I_{{\text{ref}}} }} \cdot \frac{{B1_{{\text{ref}}} }}{{B1_{{\text{organ}}} }} \cdot \frac{{{\text{sin}}(\alpha ~ \cdot ~B1_{{\text{ref}}} )}}{{{\text{sin}}(\alpha ~ \cdot B1_{{\text{organ}}} )}}$$

where *I*_organ_ and *I*_ref_ represent the image intensities in the drawn organ and phantom regions in the high-resolution ^23^Na image, whilst B1_ref_ and B1_organ_ represent the corresponding normalised intensities in the drawn regions of the *B*_1_ map. *C*_ref_ is the total sodium concentration of the agar gel phantom used as a reference (80 mM in our study). In this way, the uncorrected TSC is derived from the first two terms of Eq. (2), the receive *B*_1_ correction is applied in the third term, and the transmit *B*_1_ correction applied in the final term. The nominal flip angle α in the high-resolution data set was 70°.

Estimates of TSC from all regions of interest presented in this work have been presented as concentrations in mmol per litre of wet tissue. Absolute quantification of TSC (which would include relaxation corrections) was not performed, although relaxation bias for longitudinal *T*_1_ relaxation and the long component of *T*_2_* relaxation was assumed to be small based on the repetition time and echo time of RF pulses used in signal acquisition (TR = 100 ms » *T*_1_ and TE = 0.705 ms « *T*_2_*_long_;. More information on relaxation bias estimation can be found in Tables S1 and S2 in Section 3 of the Supporting Information (SI).

### ^23^Na *T*_2_* relaxation time constant calculation

Three-dimensional maps of the *T*_2_* relaxation time constant were calculated from the low-resolution 3D cones trajectory variable TE series in each of the healthy volunteers by taking the negative natural logarithm of the signal intensity at echo times from 2 to 16 ms and performing a linear fit (the first two echo times were not used for analysis, see Figure S4 in the Supporting Information). Thresholding was again performed relative to 10% of the maximum observed signal intensity in the shortest echo time series (TE = 0.705 ms), with intensities below this value set to zero.

For the purposes of this work, estimates of the long *T*_2_* components only were determined for each organ or structure via log-linear fitting. A comparison of mono-exponential, bi-exponential and log-linear estimation of *T*_2_* in this body of work is included in Section 4 of the Supporting Information (SI).

### Region drawing

Regions of interest were drawn on a single axial slice within MATLAB using the *roipoly* function to create logical masks over the structure or organ in both data sets, with the mean intensity values in these drawn regions only being used in the TSC and long-component ^23^Na *T*_2_* calculations. Slice selection was performed manually for each organ and volunteer by visually identifying a slice with minimal artifacts and/or partial volume effects from other nearby structures and void spaces. Regions drawn were as follows: phantoms, kidneys, CSF, liver, gallbladder, spleen, aorta, and inferior vena cava (IVC).

**Fig. 2:**
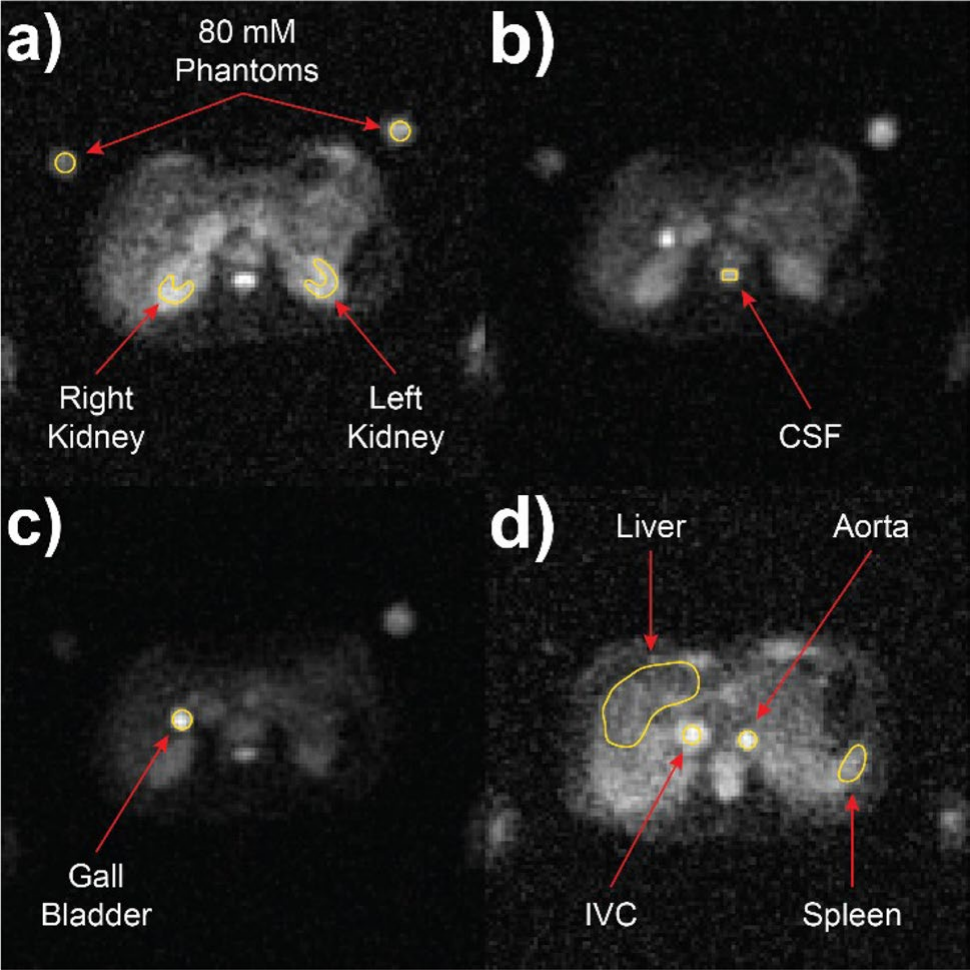
**a** Example images acquired from high-resolution 3D conical trajectory axial slices in a female healthy volunteer (age 25–30 years), illustrating the region-drawing process in MATLAB: **a** both kidneys and both phantoms; **b** CSF; **c** gallbladder; **d** spleen, liver, aorta, and inferior vena cava (IVC)

As discussed above, the TSC calculation required the drawing of regions within both the high-resolution and low-resolution, DAM data sets to perform *B*_1_ non-uniformity correction. For the long-component *T*_2_* calculation, regions drawn mirrored those used in the *B*_1_ calculations. Regions were drawn in the image series acquired using the highest flip angle for DAM and the shortest TE for *T*_2_* to maximise SNR. Regions were drawn of the approximate size, shape, and location as those shown in Fig. 2. In addition, a single large region was drawn outside of the body habitus to perform signal-to-noise ratio (SNR) measurements of uncorrected high-resolution ^23^Na signal intensity.

## Results

A total of 19 healthy volunteers (12 male, 7 female) underwent imaging for the purposes of this study.

### Double-angle method (DAM) *B*_1_ mapping

**Fig. 3:**
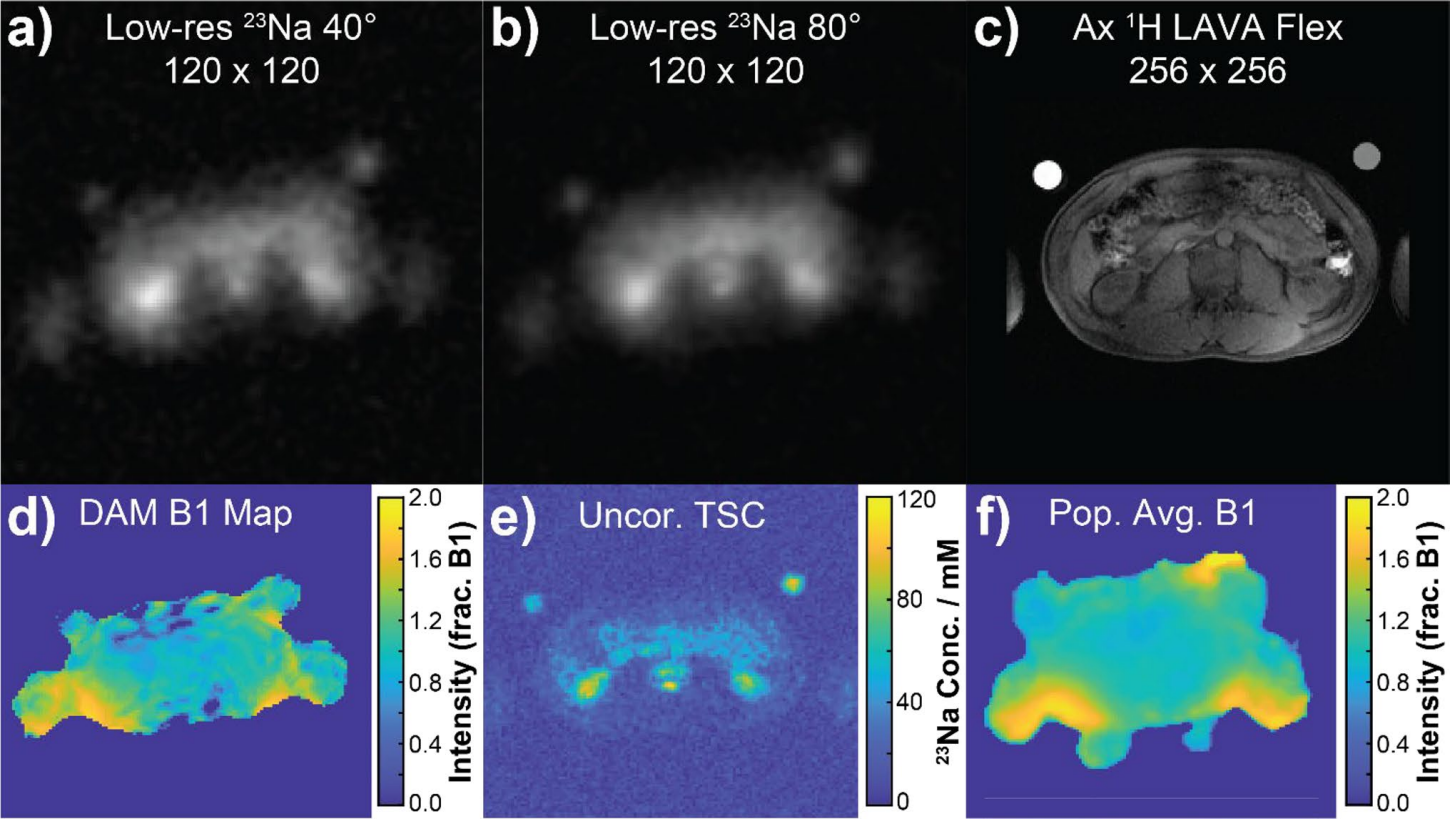
Example sodium images. **a** Low-resolution ^23^Na signal intensity image at a 40° flip angle; **b** low-resolution ^23^Na signal intensity image at an 80° flip angle; **c** fused fat/water anatomical ^1^H image (acquisition FOV = 40 cm, scaled to match other displays); **d** relative B_1_ intensity map calculated using the double-angle method; **e** high-resolution uncorrected ^23^Na signal intensity map; **f** population average estimate *B*_1_ intensity map. All images were acquired from an axial slice through the centre of the abdomen. Data presented in display (**f)** represents the median *B*_1_ across all volunteers where DAM *B*_1_ data were acquired; all other displays present data from a single healthy male volunteer (age 25–30 years) exclusively. Circles visible in the upper-left and upper-right sides of the FOV correspond to the suspended 80 mM agar NaCl phantoms

Example low-flip and high-flip ^23^Na images are presented in Fig. 3a, b, respectively, along with a corresponding ^1^H anatomical image of summed water and fat shown in Fig. 3c. The resulting *B*_1_ map created from these low-resolution ^23^Na images is shown in Fig. 3d, with the population average estimate *B*_1_ map utilised for subjects without DAM acquisition series shown in Fig. 3f). Finally, a high-resolution ^23^Na TSC map was generated before applying *B*_1_ correction, as presented in Fig. 3e. All images shown were acquired from a central axial abdominal slice. The population average estimate *B*_1_ map shown in Fig. 3f includes data from all volunteers, where DAM *B*_1_ data were acquired; all other images were acquired in a single healthy male volunteer (age 25–30 years).

Mean SNR of uncorrected ^23^Na signal intensity per-region across the healthy volunteer population was highest in the gall bladder (33 ± 15), followed by the CSF (30 ± 4) and kidneys (left: 23 ± 5, right: 26 ± 5). SNR was comparable in the aorta (21 ± 3) and IVC (22 ± 4). The lowest SNR values from uncorrected ^23^Na signal intensity images were seen in the spleen (13 ± 3) and liver (11 ± 3). SNR values for individual subjects by region can be found in Table S3 of the Supporting Information.

### Total sodium concentration

**Fig. 4:**
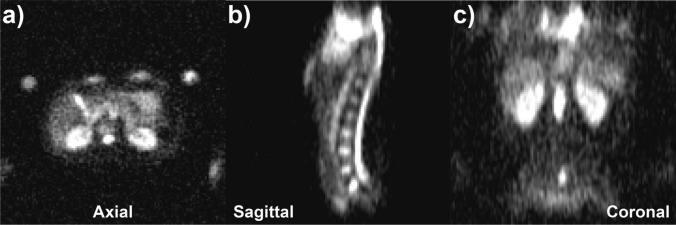
^23^Na signal intensity maps in axial, sagittal and coronal planes demonstrating sodium content in some major organs and fluid-filled regions of interest in a healthy male volunteer (age 30–35 years)

**Fig. 5:**
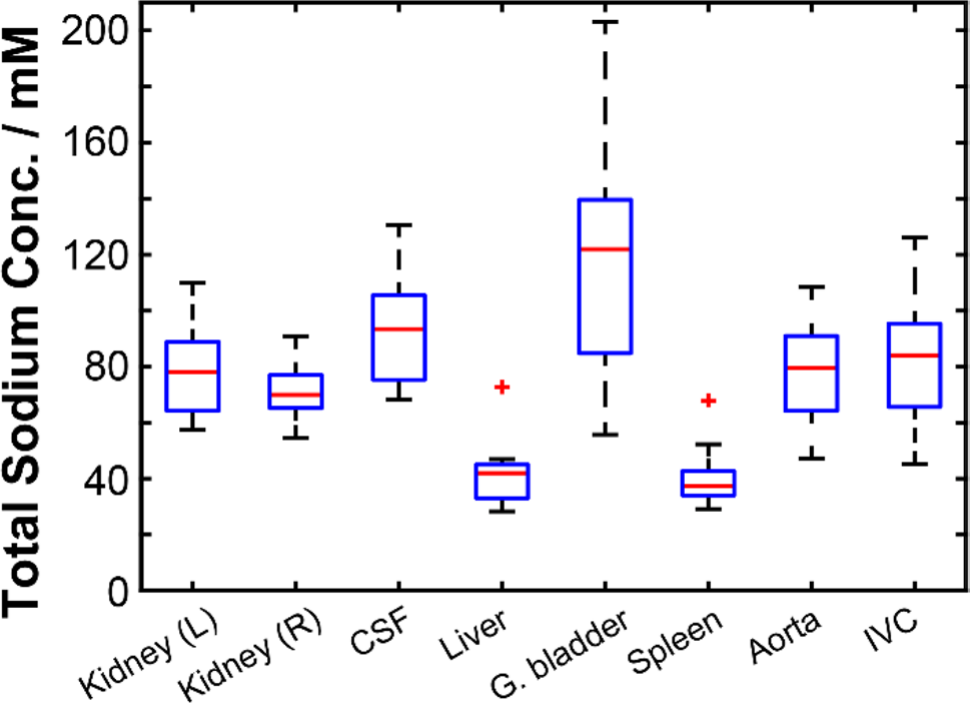
Total ^23^Na concentration box-and-whisker plots illustrating the median (red line), interquartile range (blue box) and maximum extent of non-outlier data points (dashed black lines) for each of the regions of interest interrogated. Whiskers were defined as having a maximum extent of 1.5 times the IQR for each ROI. Note that the values reported for small structures are likely underestimated relative to true concentrations reported in the literature due to partial volume effects

Intensity maps of acquired ^23^Na signal through a healthy male volunteer (age 30–35 years) are shown in Fig. 4. As discussed previously, the mean total sodium content in each organ was not determined directly from the TSC map due to non-linear image distortion between the high-resolution and DAM *B*_1_^23^Na series. *B*_1_-corrected ^23^Na TSC values were calculated independently for each organ or structure in all subjects, and are recorded in Table S3 of the Supporting Information (SI). Median ^23^Na TSC values and their associated interquartile ranges are presented in Fig. 5. For all values quoted below, the uncertainty is expressed as the population standard deviation.

Across the 19 healthy volunteers, the ^23^Na TSC was highest in the gallbladder with a mean value of 122 ± 42 mM, followed by the CSF, where the estimated mean TSC was calculated to be 94 ± 18 mM. High standard deviation of TSC was also observed in other small, fluid-filled structures, such as the aorta and IVC, where mean TSC values were 78 ± 16 mM and 83 ± 21 mM, respectively. In the kidneys, the coefficient of variation in measured total sodium concentration was somewhat reduced: 79 ± 15 mM and 71 ± 9 mM TSC in the left and right kidneys, respectively. The liver and spleen demonstrated a low sodium content and correspondingly high coefficients of variations, with observed TSC values of 41 ± 10 mM in the liver and 40 ± 9 mM, respectively.

### ^23^Na *T*_2_^*^ relaxation time constant

**Fig. 6:**
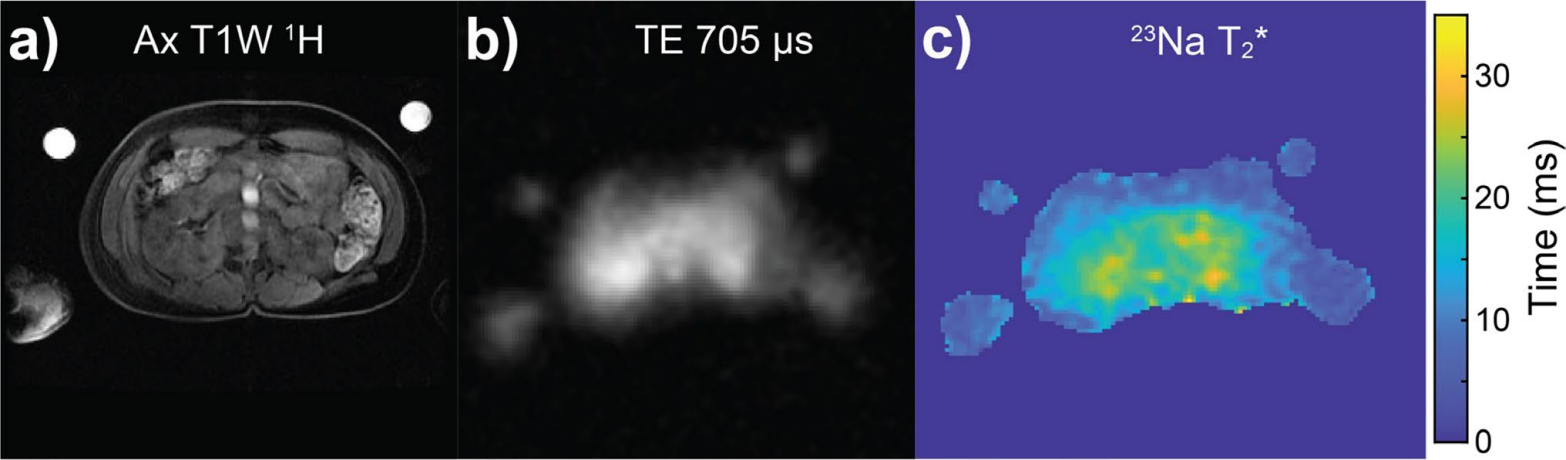
**a** Anatomical ^1^H *T*_1_-weighted image through a central axial slice centred on the kidneys for a healthy female volunteer (age 35–40 years). A corresponding axial low-resolution ^23^Na image from the series with the shortest TE (0.705 ms, not used for long-component *T*_2_* estimation) is shown in display b), whilst a single axial slice map of long-component.^23^Na *T*_2_* is shown in display c)

**Fig. 7:**
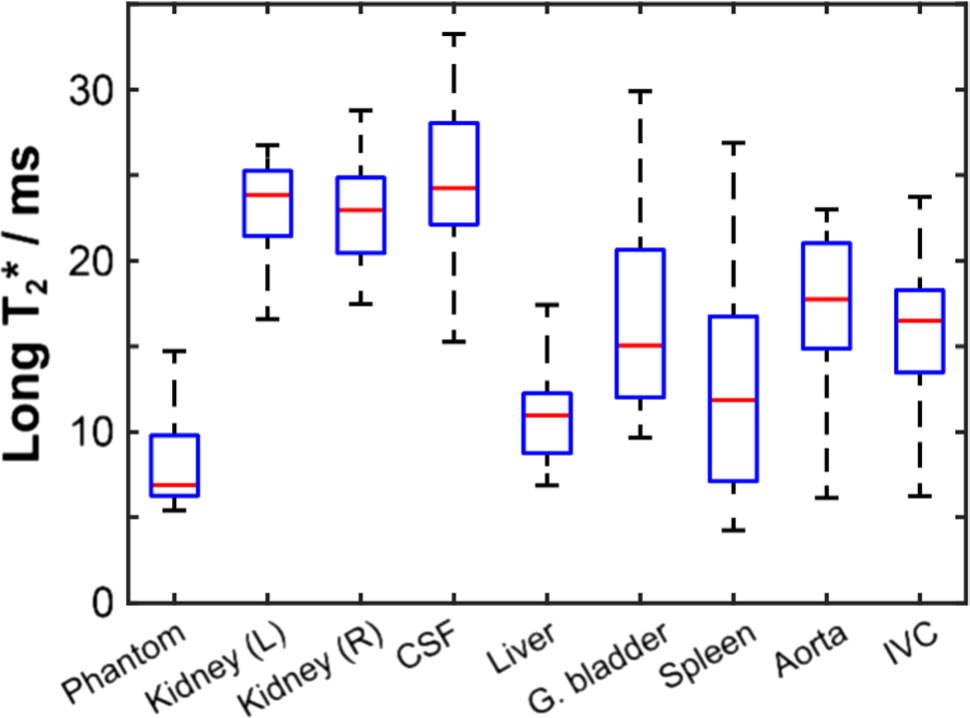
^23^Na long-component *T*_2_* relaxation time constant box-and-whisker plots illustrating the median (red line), interquartile range (blue box) and maximum extent of non-outlier data points (dashed black lines) for each of the regions of interest interrogated. Whiskers were defined as having a maximum extent of 1.5 times the IQR for each ROI

An example ^23^Na long *T*_2_* quantitative map across a single central slice centred on the kidneys from a healthy female volunteer (age 35–40 years) is presented in Fig. 6c, alongside the corresponding shortest-TE image in Fig. 6b for comparison. Median long-component ^23^Na *T*_2_* values and their associated interquartile ranges are presented in Fig. 7. Numerical ^23^Na *T*_2_* values calculated for each organ and structure in all healthy volunteers are displayed in Table S4 of the Supporting Information (SI).

Mean long-component *T*_2_* values were highest in the kidneys: 23 ± 3 ms in both cases. Relaxation was observed to be significantly more rapid in the liver with a moderate coefficient of variation (11 ± 3 ms). Whilst exhibiting a higher average long-component *T*_2_* value than the liver, intra-population variation was similarly modest in the blood: 17 ± 4 ms in the aorta and 16 ± 4 ms in the IVC. Long-component *T*_2_* relaxation time was observed to be highest in the CSF at 25 ± 5 ms. As with the B_1_-corrected TSC calculations, the most significant intra-population variation in the long-component *T*_2_* value was typically observed in the gallbladder (16 ± 6 ms), as well as the spleen (10 ± 4 ms).

## Discussion

### Double-angle method (DAM) B_1_ mapping

The uncorrected ^23^Na TSC map through a central axial slice shown in display 3f shows the sharp spatial resolution of the two agar gel phantoms, both kidneys and the CSF and spinal cord, facilitating easy region drawing within MATLAB and quantification of mean ^23^Na signal on a per-region basis, as discussed in the following section.

The *B*_1_ maps presented in Fig. 3d, f demonstrate that most organs investigated in this study are within a central region of the abdomen, where *B*_1_ variation in this coil was relatively small: mean fractional *B*_1_ value = 0.97 ± 0.07 in the population average *B*_1_ map. The most significant *B*_1_ variation was localised to structures near the rungs of the birdcage *T*/*R* body coil, with maximum fractional intensities as high as double those seen in the central region. Three-plane maps of median fractional *B*_1_ and accompanying standard deviation across the *N* = 17 healthy volunteers, where DAM *B*_1_ maps were acquired can be found in Figure S5 of the Supporting Information (SI).

In addition to the large variation in B_1_ intensity near the coil rungs which made the correction estimates less reliable, the displacement of the agar gel phantoms used for TSC calibration towards the edge of the FOV could significantly impact TSC calculation due to the prevalence of image distortions inherent in the cones sequence at the FOV edge. Whilst not observed in the healthy volunteer population, to avoid any potential future bias when imaging large participants, this observation led to the inclusion of two additional identical phantoms beneath the patient support, displaced either side of the bed centre. These additional, fixed phantoms could be used as a point of reference in the event of the suspended phantoms yielding poor SNR.

### Total sodium concentration

Estimated TSC values in the gall bladder (122 ± 42 mM) and CSF (94 ± 18 mM) were lower than established reports in the literature of ~145 mM sodium concentration in bile [50] and 151 mM in CSF in humans[51] as measured via ion chromatography and those observed in a previous quantification via ^23^Na-MRI [52]. This discrepancy, as well as high standard deviation of TSC values amongst the healthy volunteer population, was theorised to arise from partial volume effects due to the small region size, since this prior study was conducted using sagittal and coronal plane imaging which presents a significantly larger potential region of interest for CSF than in the axial plane used in this work. Use of a higher spatial resolution would reduce the effects of partial volume errors, but this naturally increases scan time and may impact clinical tolerability.

In contrast, values in the blood vessels were in reasonable agreement with those expected based on a normal extracellular sodium concentration range for healthy adults of 135–145 mM as measured in blood serum [53], coupled with a typical haematocrit fraction or red blood cell volume of ~40–45% in the total blood volume [54]. The difference in sodium content within the aorta and IVC was not statistically significant at a 5% level of significance (aorta = 78 ± 16 mM, IVC = 83 ± 21 mM, *p* = 0.052).

TSC values within the kidneys (left = 79 ± 15 mM, right = 71 ± 9 mM) were around 20 mM lower than those estimated in previous in vivo ^23^Na-MRI assessment of the medulla [43] and whole-kidney TSC [55], but were approximately 20 mM higher than those estimated elsewhere using a FLORET B_1_ non-uniformity correction method [56]. The difference in TSC values observed between the left and right kidneys across our healthy control group was found to be statistically significant at a 5% level of significance (*p* = 0.029). This unexpected result likely arises from small imperfections in the B_1_ correction applied using our low spatial resolution DAM image series. The variation in B_1_ is more pronounced near to the kidneys due to proximity of the coil rungs, as visible in Fig. 3f.

The relatively similar standard deviation values for the liver and spleen indicates that they may be primarily driven by the noise floor. The total sodium concentration values in the liver (41 ± 10 mM) were in agreement with previously reported studies in healthy rats [57–59], but substantially greater than those observed previously in human studies undertaken using surface coils [60]. Flame photometric studies in Dahl rats similarly identified low splenic sodium content relative to other organs [57] (40 ± 9 mM in this work).

### ^23^Na *T*_2_^*^ relaxation time constant

^23^Na *T*_2_* values from the different abdominal regions generally fell within the range of previously reported long ^23^Na *T*_2_* values [9, 61].

The low observed intra-population standard deviation of long-component *T*_2_* in the kidneys was expected for large organs with high relative ^23^Na signal intensity, owing to reduced variability resulting from partial volume effects. Measurements of ^23^Na *T*_2_* in the kidneys were in agreement with previous reports of 21 ± 1 ms at 3 T [43] and 7 T [42, 62]. Unlike the renal TSC values, the difference in *T*_2_* relaxation times observed between the left and right kidneys was not affected by B_1_ variation, and was not statistically significant at a 5% level of significance (left kidney = 23.2 ± 2.5 ms, right kidney = 22.7 ± 3.1 ms, *p* = 0.644).

The relatively short *T*_2_* values observed in the liver (10.7 ± 2.7 ms) are in agreement with previous ^1^H-MRI studies which identified a significantly increased component of short-*T*_2_* tissue in the liver relative to long-*T*_2_* counterparts [63–65], but are lower than those reported in rat liver [66]. This observation is thought to arise predominantly from the high content of paramagnetic iron within the liver, which has the effect of increasing ^23^Na relaxation rates [67, 68].

Observations of ^23^Na *T*_2_* in the blood were contrary to the expectations that both the rate of relaxation and the intra-population variation would be higher due to partial volume effects and the influence of fluid flow given the spatial resolution used for acquisition [69–71]. Oxygenated (aorta) and deoxygenated (IVC) blood relaxation rates in vivo were observed to be statistically different at a 5% level of significance (aorta = 17.3 ± 4.2 ms, IVC = 15.7 ± 4.0 ms, *p* = 0.013). This observation was expected considering the different magnetic properties of oxyhaemoglobin (diamagnetic) and deoxyhaemoglobin (weakly paramagnetic), as demonstrated by the higher signal intensity of oxygenated blood in *T*_2_*-weighted BOLD fMRI [72, 73].

Fluid motion effects on *T*_2_* relaxation in the CSF were not considered to be significant due to the slower rate of CSF motion relative to blood [74] and compared to the spatial imaging resolution employed here. However, the low spatial resolution may explain why the calculated values (24.6 ± 4.6 ms in this work) were significantly below those reported in studies of the brain at 7 T [75].

^23^Na *T*_2_* estimates in the gall bladder were limited by the small region size, whereas quantification in the spleen suffered from an inherently low relative ^23^Na signal intensity. Observation of the shortest long-component *T*_2_* values in the spleen is consistent with reports in a murine model comparing relaxation properties with that of the heart and liver [76], although in that study, a statistically significant difference between spleen and liver relaxation rates was noted, which is not observed here (gall bladder = 15.8 ± 6.0 ms, spleen = 10.3 ± 4.2 ms, *p* = 0.188). Human studies of ^1^H transverse relaxation rates also highlighted faster relaxation in the liver than in the spleen [77] which has not been observed for ^23^Na.

A comparison of results observed in this work with those published elsewhere in the literature is summarised in Table 1 for reference.

**Table 1:** Comparison of calculated TSC and *T*_2_* relaxation values to previously published work

Region	Study	Population	Value(s) reported	Notes
Total sodium concentration/mM				
Kidneys	Grist 2020 [55]	12 (11 M/1F)	94 ± 5, 91 ± 6	Two-site repeatability study, whole kidney measurement
	Haneder 2013 [43]	50 (30 M/20F)	99 ± 18	Medulla measurement only
	Vaeggemose 2023 [56]	5 (3 M/2F)	53 ± 27	FLORET B_1_ correction
CSF	Çavuşoğlu 2022 [52]	5 (2 M/3F)	148 ± 7	
Liver	Hooper 1976 [58]	18 (9 M/9F)	37 ± 3	Quantification in rats using flame photometry
	Tsunooka 1997 [57]	8 (all M)	33 ± 1	Quantification in rats using flame photometry
	James 2015 [60]	3 (2 M/1F)	20 ± 1	2 × surface coil array, B_1_ correction via large homogeneous phantom
Gall bladder	Furey 1966 [50]	208 (76 M/132F)	145	Measured in patients undergoing surgery
Spleen	Tsunooka 1997 [57]	8 (all M)	25 ± 1	Quantification in rats using flame photometry
*T*_2_* relaxation time/ms				
Kidneys	Haneder 2014 [42]	8 (4 M/4F)	21 ± 1	Medulla value, obtained at 7 T
	Haneder 2013 [43]	6 (2 M/4F)	21 ± 1	Mono-exponential fitting
CSF	Nagel 2011 [75]	3 (Unspecified)	56 ± 4	Mono-exponential fitting w/ 7 T head birdcage coil, 5.5 × 5.5 × 5.5 mm^3^

## Conclusions

We have demonstrated the potential for acquisition of ^23^Na signal intensity across a large proportion of the abdomen using ^23^Na-MRI and a large-FOV sodium-tuned *T*/*R* body coil with good signal-to-noise ratio in a clinically acceptable timespan. Subsequent estimation of DAM *B*_1_-corrected total sodium concentration and the long-component of the transverse ^23^Na *T*_2_* relaxation time in a variety of abdominal organs and fluid-filled structures has been demonstrated. This work will serve as a baseline to evaluate pathological changes in conditions such as hypertension and for tumour characterisation. It is hoped that the development of ^23^Na-MRI as a biomarker for tissue sodium content and dynamics may in the future inform clinical management by reducing the need for invasive biopsy techniques.

Two identical 80 mM agar gel sodium chloride phantoms were used to estimate TSC in this work to serve as a reliability test, since differences in phantom location can result in variation in local B_1_, and may also experience variation in RF artifacts. In addition, phantoms may dry out over time, increasing their effective sodium concentration. An alternative method for estimation of ^23^Na TSC which has been demonstrated in studies in the leg [78] and intervertebral discs [52] and could be applied to future work makes use of multiple agar gel phantoms of different concentrations and performing a linear fit through the ^23^Na signal intensities of each phantom and the organ or structure of interest. Presumably, as in these studies, the phantom arrangement should also occupy a fixed position within the coil, such as underneath the patient, to make phantom *B*_1_ calibration measurements more consistent, and phantoms could also be larger in size to facilitate easier region drawing. This may be particularly important in larger patients, as the suspended-phantom arrangement utilised in this work may be less reliable when phantoms are displaced close to the rungs of the coil or the edge of the field-of-view, but at the same time should not impede patient comfort. Estimation of ^23^Na *T*_2_* measurements in fluid-filled regions might be made more reliable using cardiac-triggered acquisition to suppress the effects of fluid motion, but this would have the consequence of significantly increasing scan durations.

Analysis of anatomical variation in TSC and correlations with other MR-based metrics could be facilitated by automated registration of anatomical ^1^H, low-resolution DAM *B*_1_ and uncorrected high-resolution TSC images. This may be made easier with the use of machine-learning or deep-learning registration techniques, to overcome the difficulties presented by image rotations and distortions prevalent with conical trajectories possessing long readout lengths.

## Supplementary Information

Additional methodological descriptions of B_1_ median filtering, sodium cones non-linear image distortions and *T*_2_* relaxation time estimation via log-linear and exponential fitting. Full tabulated measurements of B_1_-corrected total sodium concentration and long-component ^23^Na *T*_2_* in each region and healthy volunteer are provided. Imaging data and analysis scripts used in MATLAB are not included but will be shared upon request.

Supplementary file1 (DOCX 2029 KB)

## Data Availability

The data that support the findings of this study are available from the corresponding author upon reasonable request. Data are located in controlled access data storage at The University of Cambridge.

## References

[1] Overman RR, Davis AK (1947) The application of flame photometry to sodium and potassium determinations in biological fluids. J Biol Chem 168(2):641–649 20238620

[2] MacDonald NF, Williams PZ, Burton JI, Batsakis JG (1981) Sodium and potassium measurements: direct potentiometry and flame photometry. Am J Clin Pathol 76(4 Suppl):575–577 7282643

[3] Kricka LJ, Park JY (2014) Assay principles in clinical pathology. In: McManus LM, Mitchell RN (eds) Pathobiology of human disease. Academic Press, San Diego, pp 3207–3221

[4] Castle-Kirszbaum M, Kyi M, Wright C, Goldschlager T, Danks RA, Parkin WG (2021) Hyponatraemia and hypernatraemia: disorders of water balance in neurosurgery. Neurosurg Rev 44(5):2433–2458 33389341 10.1007/s10143-020-01450-9

[5] Dipietro ES, Bashor MM, Stroud PE, Smarr BJ, Burgess BJ, Turner WE, Neese JW (1988) Comparison of an inductively coupled plasma-atomic emission spectrometry method for the determination of calcium, magnesium, sodium, potassium, copper and zinc with atomic absorption spectroscopy and flame photometry methods. Sci Total Environ 74:249–262 3222695 10.1016/0048-9697(88)90141-6

[6] Tashiro M, Tursun P, Konishi M (2005) Intracellular and extracellular concentrations of Na+ modulate Mg2+ transport in rat ventricular myocytes. Biophys J 89(5):3235–3247 16085772 10.1529/biophysj.105.068890 PMC1366819

[7] Lo CJ, Leake MC, Berry RM (2006) Fluorescence measurement of intracellular sodium concentration in single *Escherichia coli* cells. Biophys J 90(1):357–365 16227503 10.1529/biophysj.105.071332 PMC1367033

[8] Akbari A, Lemoine S, Salerno F, Marcus TL, Duffy T, Scholl TJ, Filler G, House AA, McIntyre CW (2022) Functional sodium MRI helps to measure corticomedullary sodium content in normal and diseased human kidneys. Radiology 303(2):384–389 35133199 10.1148/radiol.211238

[9] Ouwerkerk R (2011) Sodium MRI. In: Modo M, Bulte JWM (eds) Magnetic resonance neuroimaging: methods and protocols. Humana Press, Totowa, pp 175–201

[10] Strazzullo P, D’Elia L, Kandala N-B, Cappuccio FP (2009) Salt intake, stroke, and cardiovascular disease: meta-analysis of prospective studies. BMJ 339:b4567 19934192 10.1136/bmj.b4567 PMC2782060

[11] Whelton PK, Appel LJ, Sacco RL, Anderson CAM, Antman EM, Campbell N, Dunbar SB, Frohlich ED, Hall JE, Jessup M, Labarthe DR, MacGregor GA, Sacks FM, Stamler J, Vafiadis DK, Horn LVV (2012) Sodium, blood pressure, and cardiovascular disease. Circulation 126(24):2880–2889 23124030 10.1161/CIR.0b013e318279acbf

[12] Nerbass FB, Pecoits-Filho R, McIntyre NJ, McIntyre CW, Taal MW (2015) High sodium intake is associated with important risk factors in a large cohort of chronic kidney disease patients. Eur J Clin Nutr 69(7):786–790 25293433 10.1038/ejcn.2014.215

[13] Ouwerkerk R, Bleich KB, Gillen JS, Pomper MG, Bottomley PA (2003) Tissue sodium concentration in human brain tumors as measured with 23Na MR imaging. Radiology 227(2):529–537 12663825 10.1148/radiol.2272020483

[14] Ouwerkerk R, Jacobs MA, Macura KJ, Wolff AC, Stearns V, Mezban SD, Khouri NF, Bluemke DA, Bottomley PA (2007) Elevated tissue sodium concentration in malignant breast lesions detected with non-invasive 23Na MRI. Breast Cancer Res Treat 106(2):151–160 17260093 10.1007/s10549-006-9485-4

[15] Madelin G, Regatte RR (2013) Biomedical applications of sodium MRI in vivo. J Magn Reson Imaging 38(3):511–529 23722972 10.1002/jmri.24168 PMC3759542

[16] Hu R, Kleimaier D, Malzacher M, Hoesl MAU, Paschke NK, Schad LR (2020) X-nuclei imaging: current state, technical challenges, and future directions. J Magn Reson Imaging 51(2):355–376 31102340 10.1002/jmri.26780

[17] Zaric O, Juras V, Szomolanyi P, Schreiner M, Raudner M, Giraudo C, Trattnig S (2021) Frontiers of sodium MRI revisited: from cartilage to brain imaging. J Magn Reson Imaging 54(1):58–75 32851736 10.1002/jmri.27326 PMC8246730

[18] Gast LV, Platt T, Nagel AM, Gerhalter T (2023) Recent technical developments and clinical research-applications of sodium (23Na) MRI. Prog Nucl Magn Reson Spectrosc 138–139:1–51 38065665 10.1016/j.pnmrs.2023.04.002

[19] Steidle G, Graf H, Schick F (2004) Sodium 3-D MRI of the human torso using a volume coil. Magn Reson Imaging 22(2):171–180 15010109 10.1016/j.mri.2003.08.007

[20] Wetterling F, Corteville DM, Kalayciyan R, Rennings A, Konstandin S, Nagel AM, Stark H, Schad LR (2012) Whole body sodium MRI at 3T using an asymmetric birdcage resonator and short echo time sequence: first images of a male volunteer. Phys Med Biol 57(14):4555–4567 22722731 10.1088/0031-9155/57/14/4555

[21] Bangerter NK, Kaggie JD, Taylor MD, Hadley JR (2016) Sodium MRI radiofrequency coils for body imaging. NMR Biomed 29(2):107–118 26417667 10.1002/nbm.3392

[22] Malzacher M, Kalayciyan R, Konstandin S, Haneder S, Schad LR (2016) Sodium-23 MRI of whole spine at 3 Tesla using a 5-channel receive-only phased-array and a whole-body transmit resonator. Z Med Phys 26(1):95–100 25891846 10.1016/j.zemedi.2015.03.008

[23] Platt T, Umathum R, Fiedler TM, Nagel AM, Bitz AK, Maier F, Bachert P, Ladd ME, Wielpütz MO, Kauczor HU, Behl NGR (2018) In vivo self-gated (23) Na MRI at 7 T using an oval-shaped body resonator. Magn Reson Med 80(3):1005–1019 29427389 10.1002/mrm.27103

[24] Anisimov NV, Sadykhov EG, Pavlova OS, Fomina DV, Tarasova AA, Pirogov YA (2019) Whole body sodium MRI at 0.5 Tesla using surface coil and long echo time sequence. Appl Magn Reson 50(10):1149–1161

[25] Boehmert L, Kuehne A, Waiczies H, Wenz D, Eigentler TW, Funk S, von Knobelsdorff-Brenkenhoff F, Schulz-Menger J, Nagel AM, Seeliger E, Niendorf T (2019) Cardiorenal sodium MRI at 7.0 Tesla using a 4/4 channel 1H/23Na radiofrequency antenna array. Magn Reson Med 82(6):2343–2356 31257651 10.1002/mrm.27880

[26] Malzacher M, Chacon-Caldera J, Paschke N, Schad LR (2019) Feasibility study of a double resonant (1H/23Na) abdominal RF setup at 3T. Z Med Phys 29(4):359–367 30765196 10.1016/j.zemedi.2018.12.004

[27] Milani B, Delacoste J, Burnier M, Pruijm M (2019) Exploring a new method for quantitative sodium MRI in the human upper leg with a surface coil and symmetrically arranged reference phantoms. Quant Imaging Med Surg 9(6):985–999 31367553 10.21037/qims.2019.06.08 PMC6629565

[28] Wilferth T, Mennecke A, Gast LV, Lachner S, Müller M, Rothhammer V, Huhn K, Uder M, Doerfler A, Nagel AM, Schmidt M (2022) Quantitative 7T sodium magnetic resonance imaging of the human brain using a 32-channel phased-array head coil: application to patients with secondary progressive multiple sclerosis. NMR Biomed 35(12):e4806 35892310 10.1002/nbm.4806

[29] Deen SS, Riemer F, McLean MA, Gill AB, Kaggie JD, Grist JT, Crawford R, Latimer J, Baldwin P, Earl HM, Parkinson CA, Smith SA, Hodgkin C, Moore E, Jimenez-Linan M, Brodie CR, Addley HC, Freeman SJ, Moyle PL, Sala E, Graves MJ, Brenton JD, Gallagher FA (2019) Sodium MRI with 3D-cones as a measure of tumour cellularity in high grade serous ovarian cancer. Eur J Radiol Open 6:156–162 31032385 10.1016/j.ejro.2019.04.001 PMC6477161

[30] Barrett T, Riemer F, McLean MA, Kaggie J, Robb F, Tropp JS, Warren A, Bratt O, Shah N, Gnanapragasam VJ, Gilbert FJ, Graves MJ, Gallagher FA (2018) Quantification of total and intracellular sodium concentration in primary prostate cancer and adjacent normal prostate tissue with magnetic resonance imaging. Invest Radiol 53(8):450–456 29969108 10.1097/RLI.0000000000000470

[31] Huhn K, Linz P, Pemsel F, Michalke B, Seyferth S, Kopp C, Chaudri MA, Rothhammer V, Dörfler A, Uder M, Nagel AM, Müller DN, Waschbisch A, Lee D-H, Bäuerle T, Linker RA, Haase S (2021) Skin sodium is increased in male patients with multiple sclerosis and related animal models. Proc Natl Acad Sci 118(28):e2102549118 34260395 10.1073/pnas.2102549118 PMC8285971

[32] Maifeld A, Wild J, Karlsen TV, Rakova N, Wistorf E, Linz P, Jung R, Birukov A, Gimenez-Rivera V-A, Wilck N, Bartolomaeus T, Dechend R, Kleinewietfeld M, Forslund SK, Krause A, Kokolakis G, Philipp S, Clausen BE, Brand A, Waisman A, Kurschus FC, Wegner J, Schultheis M, Luft FC, Boschmann M, Kelm M, Wiig H, Kuehne T, Müller DN, Karbach S, Markó L (2022) Skin sodium accumulates in psoriasis and reflects disease severity. J Investig Dermatol 142(1):166–178 34237339 10.1016/j.jid.2021.06.013

[33] Qirjazi E, Salerno FR, Akbari A, Hur L, Penny J, Scholl T, McIntyre CW (2020) Tissue sodium concentrations in chronic kidney disease and dialysis patients by lower leg sodium-23 magnetic resonance imaging. Nephrol Dial Transplant 36(7):1234–1243 10.1093/ndt/gfaa036 32252091

[34] Salerno FR, Akbari A, Lemoine S, Scholl TJ, McIntyre CW, Filler G (2023) Effects of pediatric chronic kidney disease and its etiology on tissue sodium concentration: a pilot study. Pediatr Nephrol 38(2):499–507 35655040 10.1007/s00467-022-05600-7

[35] Hanson P, Philp CJ, Randeva HS, James S, O’Hare JP, Meersmann T, Pavlovskaya GE, Barber TM (2021) Sodium in the dermis colocates to glycosaminoglycan scaffold, with diminishment in type 2 diabetes mellitus. JCI Insight 6(12):e145470 34003801 10.1172/jci.insight.145470 PMC8262470

[36] Kopp C, Linz P, Dahlmann A, Hammon M, Jantsch J, Müller DN, Schmieder RE, Cavallaro A, Eckardt K-U, Uder M, Luft FC, Titze J (2013) 23Na magnetic resonance imaging-determined tissue sodium in healthy subjects and hypertensive patients. Hypertension 61(3):635–640 23339169 10.1161/HYPERTENSIONAHA.111.00566

[37] Martin K, Tan S-J, Toussaint ND (2022) Magnetic resonance imaging determination of tissue sodium in patients with chronic kidney disease. Nephrology 27(2):117–125 34510658 10.1111/nep.13975

[38] Panek R, Welsh L, Dunlop A, Wong KH, Riddell AM, Koh DM, Schmidt MA, Doran S, McQuaid D, Hopkinson G, Richardson C, Nutting CM, Bhide SA, Harrington KJ, Robinson SP, Newbold KL, Leach MO (2016) Repeatability and sensitivity of T2* measurements in patients with head and neck squamous cell carcinoma at 3T. J Magn Reson Imaging 44(1):72–80 26800280 10.1002/jmri.25134 PMC4915498

[39] O’Connor JPB, Robinson SP, Waterton JC (2019) Imaging tumour hypoxia with oxygen-enhanced MRI and BOLD MRI. Br J Radiol 92(1096):1–12 (**20180642**) 10.1259/bjr.20180642 PMC6540855 30272998

[40] Robinson SP, Howe FA, Rodrigues LM, Stubbs M, Griffiths JR (1998) Magnetic resonance imaging techniques for monitoring changes in tumor oxygenation and blood flow. Semin Radiat Oncol 8(3):197–207 9634496 10.1016/s1053-4296(98)80045-3

[41] Peng Y, Luo Y, Hu X, Shen Y, Hu D, Li Z, Kamel I (2021) Quantitative T2*-weighted imaging and reduced field-of-view diffusion-weighted imaging of rectal cancer: correlation of R2* and apparent diffusion coefficient with histopathological prognostic factors. Front Oncol 11:670156 34109120 10.3389/fonc.2021.670156 PMC8180870

[42] Haneder S, Juras V, Michaely HJ, Deligianni X, Bieri O, Schoenberg SO, Trattnig S, Zbýň Š (2014) In vivo sodium (23Na) imaging of the human kidneys at 7 T: preliminary results. Eur Radiol 24(2):494–501 24081646 10.1007/s00330-013-3032-6

[43] Haneder S, Kettnaker P, Konstandin S, Morelli JN, Schad LR, Schoenberg SO, Michaely HJ (2013) Quantitative in vivo 23Na MR imaging of the healthy human kidney: determination of physiological ranges at 3.0T with comparison to DWI and BOLD. Magn Reson Mater Phys Biol Med 26(6):501–509 10.1007/s10334-013-0369-4 23475308

[44] Kaggie JD, Lanz T, McLean MA, Riemer F, Schulte RF, Benjamin AJV, Kessler DA, Sun C, Gilbert FJ, Graves MJ, Gallagher FA (2021) Combined (23) Na and (13) C imaging at 3.0 Tesla using a single-tuned large FOV birdcage coil. Magn Reson Med 86(3):1734–1745 33934383 10.1002/mrm.28772

[45] Sacolick LI, Wiesinger F, Hancu I, Vogel MW (2010) B1 mapping by Bloch–Siegert shift. Magn Reson Med 63(5):1315–1322 20432302 10.1002/mrm.22357 PMC2933656

[46] Cunningham CH, Pauly JM, Nayak KS (2006) Saturated double-angle method for rapid B1+ mapping. Magn Reson Med 55(6):1326–1333 16683260 10.1002/mrm.20896

[47] Allen SP, Morrell GR, Peterson B, Park D, Gold GE, Kaggie JD, Bangerter NK (2011) Phase-sensitive sodium B1 mapping. Magn Reson Med 65(4):1125–1130 21413078 10.1002/mrm.22700 PMC3073006

[48] Lommen J, Konstandin S, Krämer P, Schad LR (2016) Enhancing the quantification of tissue sodium content by MRI: time-efficient sodium B1 mapping at clinical field strengths. NMR Biomed 29(2):129–136 25904161 10.1002/nbm.3292

[49] Gurney PT, Hargreaves BA, Nishimura DG (2006) Design and analysis of a practical 3D cones trajectory. Magn Reson Med 55(3):575–582 16450366 10.1002/mrm.20796

[50] Furey AT (1966) Hyponatremia after choledochostomy and T tube drainage. Am J Surg 112(6):850–855 5923802 10.1016/0002-9610(66)90137-1

[51] Harrington MG, Salomon RM, Pogoda JM, Oborina E, Okey N, Johnson B, Schmidt D, Fonteh AN, Dalleska NF (2010) Cerebrospinal fluid sodium rhythms. Cerebrospinal Fluid Res 7:3 20205754 10.1186/1743-8454-7-3 PMC2822736

[52] Çavuşoğlu M, Pazahr S, Ciritsis AP, Rossi C (2022) Quantitative 23Na-MRI of the intervertebral disk at 3 T. NMR Biomed 35(8):e4733 35307881 10.1002/nbm.4733 PMC9540256

[53] Dmitrieva NI, Liu D, Wu CO, Boehm M (2022) Middle age serum sodium levels in the upper part of normal range and risk of heart failure. Eur Heart J 43(35):3335–3348 35348651 10.1093/eurheartj/ehac138 PMC10263272

[54] Billett HH (1990) Hemoglobin and hematocrit. In: Walker HK, Hall WD, Hurst JW (eds) Clinical methods: the history, physical, and laboratory examinations. Butterworths, Boston. https://pubmed.ncbi.nlm.nih.gov/21250102/21250045

[55] Grist JT, Riemer F, Hansen ESS, Tougaard RS, McLean MA, Kaggie J, Bøgh N, Graves MJ, Gallagher FA, Laustsen C (2020) Visualization of sodium dynamics in the kidney by magnetic resonance imaging in a multi-site study. Kidney Int 98(5):1174–1178 32585166 10.1016/j.kint.2020.04.056 PMC7652549

[56] Vaeggemose M, Schulte RF, Laustsen C (2023) Clinically feasible B1 field correction for multi-organ sodium imaging at 3 T. NMR Biomed 36(2):e4835 36115017 10.1002/nbm.4835 PMC10078323

[57] Tsunooka K, Morita H (1997) Effect of a chronic high-salt diet on whole-body and organ sodium contents of Dahl rats. J Hypertens 15(8):851–856 9280207 10.1097/00004872-199715080-00008

[58] Hooper G, Dick DA (1976) Nonuniform distribution of sodium in the rat hepatocyte. J Gen Physiol 67(4):469–474 1271040 10.1085/jgp.67.4.469 PMC2214917

[59] Everett JL, Day CL, Bergel F (1964) Analysis of August rat liver for calcium, copper, iron, magnesium, manganese, molybdenum, potassium, sodium and zinc*. J Pharm Pharmacol 16(2):85–90 14119546 10.1111/j.2042-7158.1964.tb07426.x

[60] James JR, Panda A, Lin C, Dydak U, Dale BM, Bansal N (2015) In vivo sodium MR imaging of the abdomen at 3T. Abdom Imaging 40(7):2272–2280 25952570 10.1007/s00261-015-0428-6 PMC6159882

[61] Riemer F, Solanky BS, Wheeler-Kingshott CAM, Golay X (2018) Bi-exponential 23Na T2* component analysis in the human brain. NMR Biomed 31(5):e3899 29480533 10.1002/nbm.3899

[62] Zbyn S, Juras V, Michaely HJ, Deligianni X, Bieri O, Schoenberg SO, Trattnig S, Haneder S (2013) Sodium t2* mapping of the human kidneys in vivo at 7 Tesla. Proc Intl Soc Mag Reson Med 21:4142

[63] Bernardino ME, Small W, Goldstein J, Sewell CW, Sones PJ, Gedgaudas-McClees K, Galambos JT, Wenger J, Casarella WJ (1983) Multiple NMR T2 relaxation values in human liver tissue. AJR Am J Roentgenol 141(6):1203–1208 6606317 10.2214/ajr.141.6.1203

[64] Westwood MA, Anderson LJ, Firmin DN, Gatehouse PD, Lorenz CH, Wonke B, Pennell DJ (2003) Interscanner reproducibility of cardiovascular magnetic resonance T2* measurements of tissue iron in thalassemia. J Magn Reson Imaging 18(5):616–620 14579406 10.1002/jmri.10396

[65] Zhu A, Hernando D, Johnson KM, Reeder SB (2019) Characterizing a short T(2) * signal component in the liver using ultrashort TE chemical shift-encoded MRI at 1.5T and 3.0T. Magn Reson Med 82(6):2032–2045 31270858 10.1002/mrm.27876 PMC6717026

[66] Bansal N, Germann MJ, Seshan V, Shires GT 3rd, Malloy CR, Sherry AD (1993) Thulium 1,4,7,10-tetraazacyclododecane-1,4,7,10-tetrakis(methylene phosphonate) as a 23Na shift reagent for the in vivo rat liver. Biochemistry 32(21):5638–5643 8504084 10.1021/bi00072a020

[67] Wood JC, Enriquez C, Ghugre N, Tyzka JM, Carson S, Nelson MD, Coates TD (2005) MRI R2 and R2* mapping accurately estimates hepatic iron concentration in transfusion-dependent thalassemia and sickle cell disease patients. Blood 106(4):1460–1465 15860670 10.1182/blood-2004-10-3982 PMC1895207

[68] Hernando D, Levin YS, Sirlin CB, Reeder SB (2014) Quantification of liver iron with MRI: state of the art and remaining challenges. J Magn Reson Imaging 40(5):1003–1021 24585403 10.1002/jmri.24584 PMC4308740

[69] Wexler L, Bergel DH, Gabe IT, Makin GS, Mills CJ (1968) Velocity of blood flow in normal human venae cavae. Circ Res 23(3):349–359 5676450 10.1161/01.res.23.3.349

[70] Stein PD, Sabbah HN, Anbe DT, Walburn FJ (1979) Blood velocity in the abdominal aorta and common iliac artery of man. Biorheology 16:249–255 508934 10.3233/bir-1979-16313

[71] Vieli A, Moser U, Maier S, Meier D, Boesiger P (1989) Velocity profiles in the normal human abdominal aorta: a comparison between ultrasound and magnetic resonance data. Ultrasound Med Biol 15(2):113–119 2658233 10.1016/0301-5629(89)90160-9

[72] Ogawa S, Lee TM, Kay AR, Tank DW (1990) Brain magnetic resonance imaging with contrast dependent on blood oxygenation. Proc Natl Acad Sci USA 87(24):9868–9872 2124706 10.1073/pnas.87.24.9868 PMC55275

[73] Whang JS, Kolber M, Powell DK, Libfeld E (2015) Diffusion-weighted signal patterns of intracranial haemorrhage. Clin Radiol 70(8):906–916 10.1016/j.crad.2015.04.006 26050534

[74] Battal B, Kocaoglu M, Bulakbasi N, Husmen G, Sanal HT, Tayfun C (2011) Cerebrospinal fluid flow imaging by using phase-contrast MR technique. Br J Radiol 84(1004):758–765 21586507 10.1259/bjr/66206791 PMC3473435

[75] Nagel AM, Bock M, Hartmann C, Gerigk L, Neumann J-O, Weber M-A, Bendszus M, Radbruch A, Wick W, Schlemmer H-P, Semmler W, Biller A (2011) The potential of relaxation-weighted sodium magnetic resonance imaging as demonstrated on brain tumors. Investig Radiol 46(9):539–547 21577129 10.1097/RLI.0b013e31821ae918

[76] Jackson LH, Vlachodimitropoulou E, Shangaris P, Roberts TA, Ryan TM, Campbell-Washburn AE, David AL, Porter JB, Lythgoe MF, Stuckey DJ (2017) Non-invasive MRI biomarkers for the early assessment of iron overload in a humanized mouse model of β-thalassemia. Sci Rep 7(1):43439 28240317 10.1038/srep43439 PMC5327494

[77] Schwenzer NF, Machann J, Haap MM, Martirosian P, Schraml C, Liebig G, Stefan N, Häring H-U, Claussen CD, Fritsche A, Schick F (2008) T2* relaxometry in liver, pancreas, and spleen in a healthy cohort of one hundred twenty-nine subjects-correlation with age, gender, and serum ferritin. Investig Radiol 43(12):854–860 19002057 10.1097/RLI.0b013e3181862413

[78] Gast LV, Baier LM, Chaudry O, Meixner CR, Müller M, Engelke K, Uder M, Heiss R, Nagel AM (2023) Assessing muscle-specific potassium concentrations in human lower leg using potassium magnetic resonance imaging. NMR Biomed 36(1):1–13 (**e4819**) 10.1002/nbm.4819 35994248

