# Supplementary material for: Quantitative 23Na magnetic resonance imaging in the abdomen at 3 T
Source: MAGMA. 2024 Jun 1;37(4):737–48. doi: 10.1007/s10334-024-01167-6 (PMC11417083; doi:10.1007/s10334-024-01167-6)
Supplement: Supplementary file 1 — Supplementary file1 (DOCX 2029 KB) [file 10334_2024_1167_MOESM1_ESM.docx]

SUPPORTING INFORMATION

for

**Quantitative ^23^Na Magnetic Resonance Imaging in the Abdomen at 3 T**

Jonathan R. Birchall^1^, Ines Horvat-Menih^1^, Joshua D. Kaggie^1^, Frank Riemer^2^, Arnold J. V. Benjamin^1^, Martin J. Graves^1^, Ian Wilkinson^3^, *Ferdia A. Gallagher^1^ and *Mary A. McLean^1^

^1^ Department of Radiology, University of Cambridge, Cambridge, United Kingdom

^2^ Mohn Medical Imaging and Visualization Centre, Department of Radiology, Haukeland University Hospital Helse Bergen, Bergen, Norway

^3^ Cambridge Cardiovascular, University of Cambridge, Cambridge, United Kingdom

* These authors contributed equally to this work

*Corresponding Author: Jonathan Birchall (jb2455@cam.ac.uk)*

**Table of Contents**

[1) B_1_ Median Filtering 3](#_Toc162524675)

[2) TSC Quantification 4](#_Toc162524676)

[3) Estimation of ^23^Na Relaxation Bias 6](#_Toc162524677)

[4) ^23^Na T_2_* Calculation from Log-Linear and Exponential Fitting 8](#_Toc162524678)

[5) Evaluation of Population Average B_1_ Mapping 10](#_Toc162524679)

[6) Total Sodium Concentration Measurements 12](#_Toc162524680)

[7) Long-Component ^23^Na T_2_* Measurements 14](#_Toc162524681)

[8) References Used in Supporting Information 15](#_Toc162524682)

# B_1_ Median Filtering

As part of the B_1_ inhomogeneity correction process for improving the accuracy of TSC quantification in our study, a 3D median filtering kernel was applied to the relative B_1_ intensity maps for each patient using the in-built MATLAB function *medfilt3*. This was done to minimize the effects of extreme local minima (dark patches observed in the B_1_ map, typically close to the centre of the coil geometry) providing an unrealistic estimate of the relative B_1_ intensity which could have led to gross over- or under-correction of TSC. A kernel of size 5 x 5 x 5 voxels was found to strike a suitable balance between performing enough smoothing to negate commonly-sized regions of low/null signal, whilst minimizing the loss of edge information from small structures. An example B_1_ map from a healthy male volunteer (age 30-35 years) both before and after application of a 3D median filter is shown below in Figure S1.


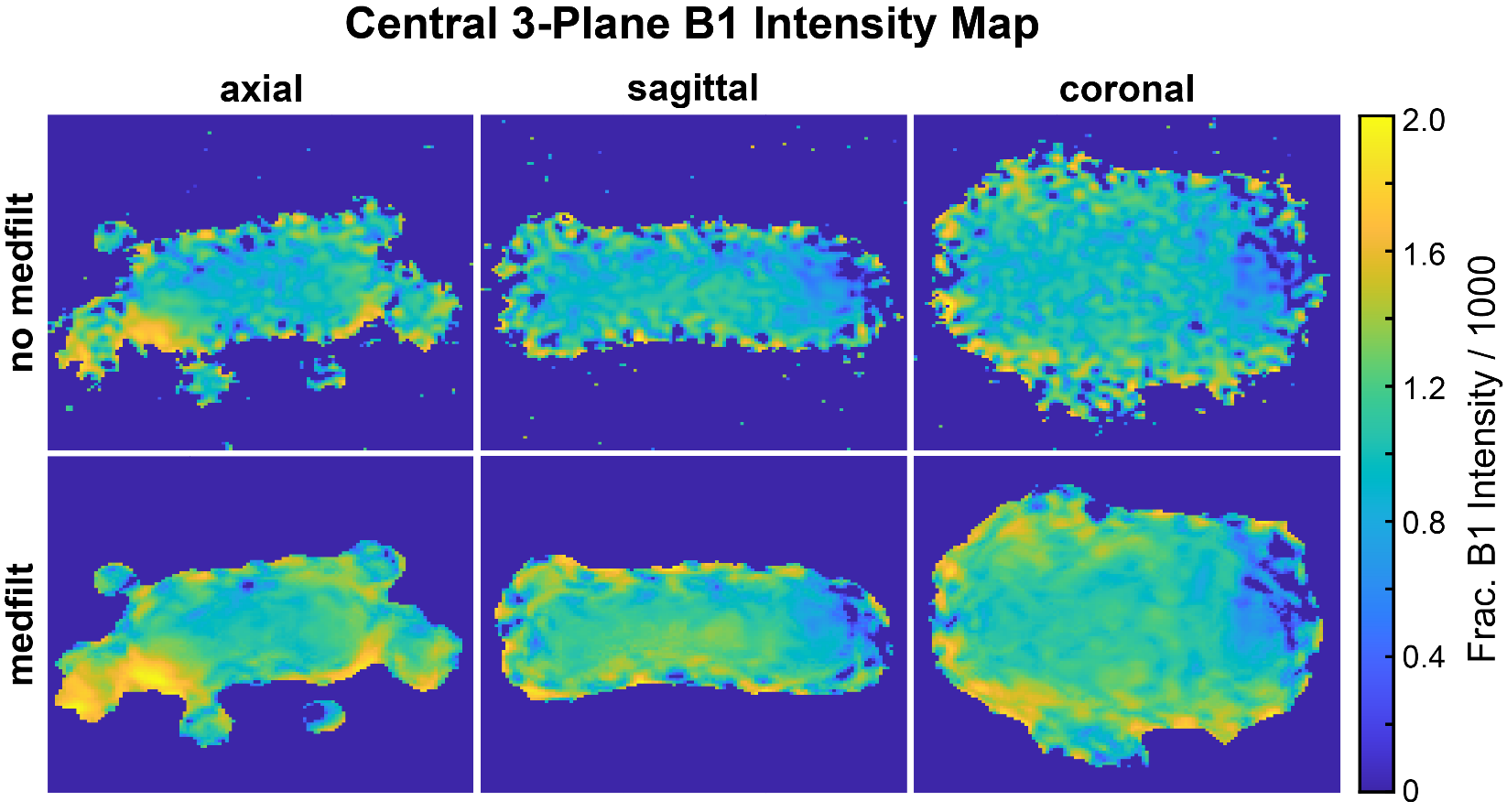


**Fig. S1** Maps of fractional normalised B_1_ intensity through central slices of a single healthy male volunteer (age 30-35 years) in the axial (left column), sagittal (central column) and coronal (right column) planes. The top row illustrates the B_1_ map as acquired using only Eq. 1 of the main text, whereas the bottom row demonstrates the same analysis whilst also including the 3D median filter (kernel size = 5). For the purposes of TSC calculation in the main study, B_1_ uniformity correction was performed using the 3D median-filtered maps

# TSC Quantification

The coronal abdominal maximum intensity projection (MIP) clearly highlights regions of high total sodium content such as the kidneys and spinal cord and gall bladder, as can be seen in Figure S2 for a healthy male volunteer (age 35-40). However, such a projection is less useful for interrogating organs and structures with lower sodium content and may struggle to resolve high sodium-containing structures in close proximity to one another, such as the intervertebral disk and surrounding CSF. To this end, axial maps from the high-resolution 3D cones series were used in the quantification of TSC for all organs and healthy volunteers.


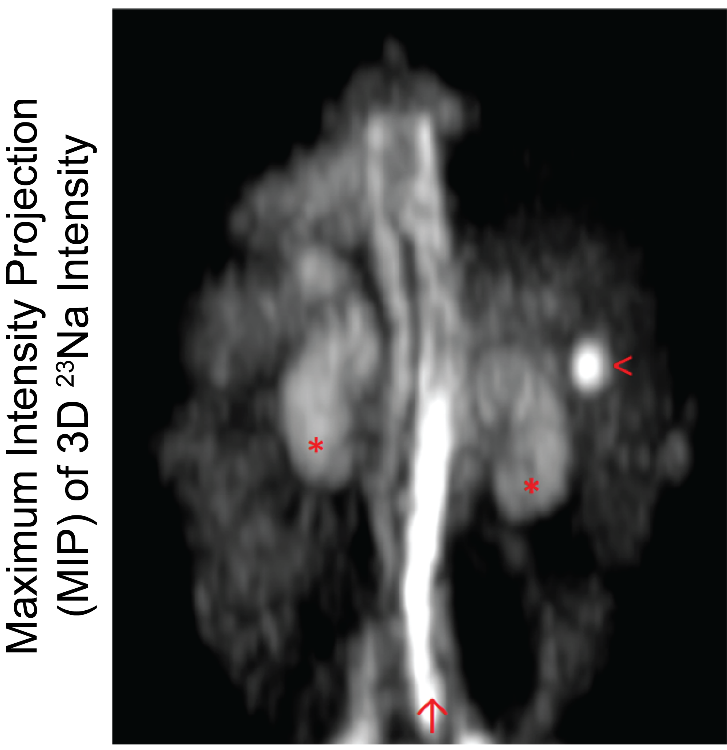


**Fig. S2** a) Coronal maximum intensity projection (MIP) reconstruction from high-resolution 3D ^23^Na-MRI data, acquired in a healthy male volunteer (age 35-40). The kidneys (asterisks), spinal cord (arrow) and gall bladder (arrowhead, right of image) demonstrate increased signal due to the high fluid content of these organs

As mentioned in the main text, B_1_-corrected maps of TSC have not been shown in this work due to differences in non-linear image distortions between high- and low-resolution cones trajectories, which made registration of anatomical ^1^H, high-resolution ^23^Na and low-resolution DAM B_1_ cones images unreliable. This effect arises due to gradient non-linearities over the large field-of-view employed in this work, contributing to a warping of anatomy at the periphery.[[1](#_ENREF_1)] A compounding issue is the difference in the duration for the two trajectories used in high and low-resolution ^23^Na imaging, which may result in image distortions similar to those observed in EPI sequences with long readouts.[[2](#_ENREF_2),[3](#_ENREF_3)] As the low-resolution DAM and the high-resolution sodium sequences had different readout lengths, and considering the comparatively high gradient slew rates used in cones imaging, the gradient distortions between them were observed to be noticeably different.

This is demonstrated in a male healthy volunteer (age 40-45 years) in Figure S3, overlaying both high-resolution ^23^Na signal intensity and low-resolution DAM B_1_ map slices in three planes with the corresponding ^1^H anatomical LAVA-Flex GRE counterparts. It can be seen from these overlays that regions of interest drawn on the high-resolution sodium images do not reliably correspond to their counterparts in the low-resolution DAM images. Because of this, B_1_-corrected TSC values were determined by drawing ROIs for each organ or fluid-filled region of interest in both the high- and low-resolution series, and determining the mean values for uncorrected TSC, receive and transmit correction factors (Equation 2 in the main text).


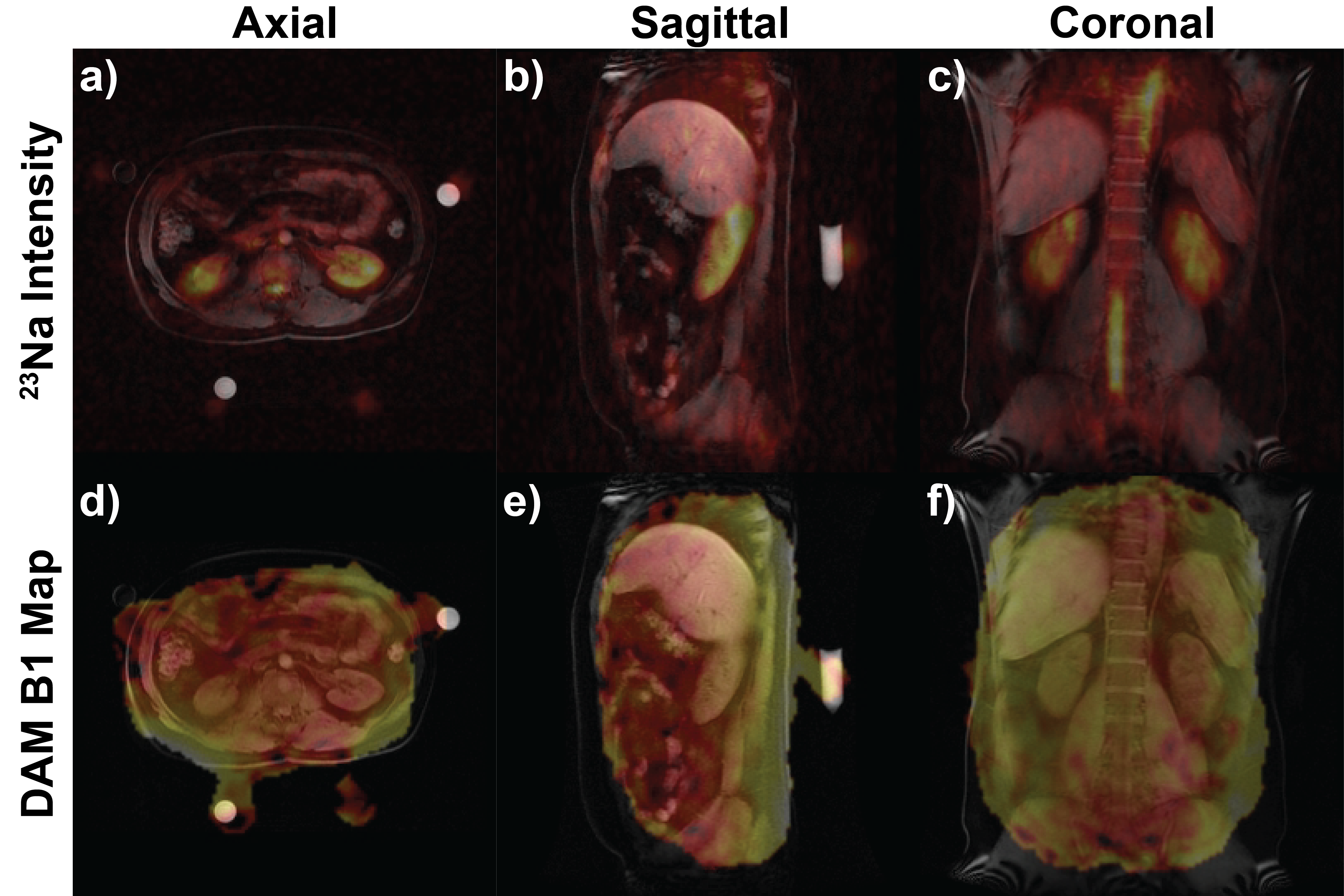


**Fig. S3** 3-Plane reconstructions of high-resolution ^23^Na-MRI cones data (top row) and low-resolution DAM B_1_ maps (bottom row) from a healthy male volunteer (age 40-45) overlaid on corresponding ^1^H anatomical images. Non-linear image distortions were more pronounced with distance from the coil isocentre in both cases, but were not consistent between the high- and low-resolution series. This is most visible by inspecting the displacement of signal originating from the agar gel phantoms seen in the axial and sagittal views. Phantoms not visible in the axial view have been identified as fat by the LAVA-Flex GRE sequence (water images only shown here)

# Estimation of ^23^Na Relaxation Bias

TSC values presented in this work have been estimated in the absence of relaxation correction required to perform absolute quantification. The fractional ^23^Na signal loss, due to T_1_ saturation, f_1_ was calculated using Equation S1 below,

$f_{1}=1-\frac{1-e^{{-TR}/{T_{1}}}}{1-\cos\left( \alpha\right)e^{{-TR}/{T_{1}}}}$ (S1)

where TR equals the repetition time of RF pulses and α represents the flip angle (100 ms and 70°, respectively in our high resolution ^23^Na-MRI 3D cones sequence). Literature values for ^23^Na T_1_ relaxation times *in vivo* of various tissues of interest to this work at a field strength of 3 T, as well as estimates for fractional signal loss (f1_organ_) in our results based on these T_1_ values are presented in Table S1 below. A measurement of T_1_ relaxation time in the two 80 mM NaCl agar gel phantoms used in this work is included for comparison, acquired using an inversion recovery (IR) sequence with the following parameters: TR = 300 ms, TE = 0.455 ms, flip angle = 70°, trajectory resolution = 9 x 9 x 9 mm, 197 transients, averages = 4, 125 kHz full receiver bandwidth, total duration = 1:28 mins per sequence, TIs = 0, 25, 50, 75, 125 ms.

Using the known fractional signal loss due to T_1_ relaxation within our phantom (f1_ref_), the positive or negative TSC bias, Δ_T1_, in organ regions was estimated using Equation S2:

$\Delta_{T1} =1-\frac{1-{f1}_{organ}}{1-{f1}_{ref}}$ (S2)

Positive bias represents an underestimation of TSC, since the tissue was more T_1_-saturated relative to the phantom and signal loss was higher, whereas negative bias represents an overestimation due to the organ being less T_1_-saturated relative to the phantom and signal loss being less significant.

| **Region** | **Study** | **T_1_ / ms** | **Notes** | **Estimated frac. signal loss, f** | **Estimated**  **TSC Bias, Δ** |
| --- | --- | --- | --- | --- | --- |
| Phantom | *This work* | 29.5 ± 1.5 |  | 1.3% | N/A |
| Kidneys | Gomolka 2019[[7](#_ENREF_7)] | 29 ± 10 |  | 1.2% | -0.1% |
| CSF | Gomolka 2019[[7](#_ENREF_7)] | 35 ± 8 |  | 2.2% | 0.9% |
| Liver | Bansal 1993[[8](#_ENREF_8)] | 33.8 ± 0.3 | Quantification in rat liver | 2.0% | 0.7% |
| Blood | Madelin 2013[[9](#_ENREF_9)] | 20 - 40 | Range of values presented in literature review | 0.25 - 3.2% | -1.0 - 1.9% |

**Table S1** Measured ^23^Na T_1_ relaxation values in phantoms in this work, measured ^23^Na T_1_ relaxation values in literature reports for organs and tissues of interest to this study at 3 T *in vivo*, and estimated fractional ^23^Na signal loss and TSC estimation bias for these tissues

Even at the upper estimate for ^23^Na T_1_ relaxation time in blood of 40 ms, fractional signal loss due to T_1_ saturation of RF pulses applied at a repetition time of 100 ms as in our experiments was estimated to be on the order of only 3%. Comparison with phantom T_1_ relaxation rates in our work indicates that correction for T_1_ relaxation bias would be on the order of 1-2% at most.

Similarly, ^23^Na signal loss due to the long component of T_2_* relaxation was also deemed to be small based on measurements made in this study. Using Equation S3 below,

$f_{2l}=1-e^{{-TE}/{T_{2}*(long)}}$ (S3)

the fractional ^23^Na signal loss due to the long component of T_2_* relaxation (f_2l_) was derived from the echo time of RF pulses supplied and the measured T_2_* of each organ or fluid-filled structure of interest. With the echo time used for the high-resolution ^23^Na cones sequence being 0.705 ms, this provided an estimated range for ^23^Na signal loss due to the long component of T_2_* relaxation being ~3% in organs with long T_2_* relaxation times (kidneys, CSF), up to 7% in the fastest-relaxing organ (spleen) and 8% in the agar gel phantom used for signal calibration.

Using the calculated mean long-component T_2_* values (see Table S4), and by combining Equations S2 and S3, the bias in estimation of mean TSC per region of interest due to the long component of T_2_* relaxation is presented in Table S2.

| **Region** | **Mean long T_2_* / ms** | **Frac. signal loss, f** | **TSC Bias, Δ** |
| --- | --- | --- | --- |
| Phantom | 8.2 | 8.24% | N/A |
| Left Kidney | 23.2 | 2.99% | -5.72% |
| Right Kidney | 22.7 | 3.06% | -5.65% |
| CSF | 24.6 | 2.83% | -5.90% |
| Liver | 10.7 | 6.38% | -2.03% |
| Gall Bladder | 15.8 | 4.36% | -4.22% |
| Spleen | 10.3 | 6.62% | -1.77% |
| Aorta | 17.3 | 3.99% | -4.63% |
| IVC | 15.7 | 4.39% | -4.19% |

**Table S2** Calculated fractional ^23^Na signal loss and TSC estimation bias due to long-component T_2_* relaxation for organs and tissues studied in this work

Given the more rapid rate of T_2_* relaxation in the agar gel phantom relative to tissue *in vivo*, all estimations of TSC relaxation bias due to the long component of T_2_* were found to be negative; i.e., effective mean TSC was slightly overestimated in all tissue regions due to slower T_2_* relaxation and loss of signal relative to the phantom. However, as shown in Table S2, this relaxation bias remains on the order of 2-6% for the regions studied in this work.

Using Equation S3 once again, we determined that the main contributing factor to ^23^Na signal loss would indeed be the short component of T_2_* relaxation. Whilst the short component was not extensively assessed in this work (see Section 4 of the SI for more details), estimates of 1-3 ms *in vivo* for abdominal tissues studied here yielded fractional ^23^Na signal loss estimates of $f_{2s}=1-e^{{-TE}/{T_{2}*(short)}}$ = 21-51% for echo times of 0.705 ms.

# ^23^Na T_2_* Calculation from Log-Linear and Exponential Fitting

Maps of the ^23^Na transverse relaxation time constant (T_2_*) across the abdomen of healthy volunteers were generated as described in Section 3.2 of the main text. As part of this process, a log-linear relationship was characterised in MATLAB between the signal intensity and the corresponding echo time. The inverse of the gradient of the resulting linear fit was taken as the value of T_2_* for each voxel. An example of such a log-linear calculation is demonstrated in a central voxel from the left kidney of a healthy female volunteer (age 35-40 years) in Figure S4a below, including R^2^ as a goodness-of-fit indicator.

Due to the quadrupolar nature of the ^23^Na nucleus, mono-exponential fitting was deemed unreliable at short echo times (TE < 2 ms)[[4](#_ENREF_4)] – see log intensities situated below the linear fit at these timepoints. A biexponential fit of the form

$y=(a*\exp\left( bx \right))+(c*\exp\left( dx \right))$ (S4)

Was attempted in MATLAB using the *fittype(‘Exp2’)* function, where the first exponential term described short-T_2_* relaxation, whilst the long-T_2_* component was defined by the second term. As shown in Figure S4b, this approach yielded short and long T_2_* components of 2.6 and 28.3 ms, respectively inside the left kidney of a single healthy female volunteer (age 35-40). These values agree well with previous studies in intact rat kidneys at 4.7 T.[[5](#_ENREF_5)] However, the small range of echo times probed and large variation in tissue relaxation properties made selecting a single start point for iterative fitting that was suitable across all voxels of the six ^23^Na signal intensity volumes unreliable. A study of biexponential T_2_ relaxation in Monte Carlo simulations and *ex vivo* imaging of bovine knee cartilage quantified the significantly higher estimation error of biexponential fitting with only 6 TEs.[[6](#_ENREF_6)] This approach was therefore not considered robust enough for application across all regions and volunteers whilst maintaining a clinically acceptable scan duration.

Since separation of the long and short T_2_* components was deemed unreliable, the long component of T_2_* was estimated instead by fitting through echo times greater than or equal to 2 ms only (the last four series in this body of work), with the caveat that the early echos included some effect of the short component. With TE = 4 ms, the remaining contribution to ^23^Na signal of fast-relaxing spins undergoing short T_2_* relaxation was estimated to be ~21% in the kidney, compared to ~87% of the original long component T_2_* ^23^Na signal—see Section 3 of the Supporting Information for commentary on the estimation of uncertainty in TSC due to relaxation bias. Whilst the ^23^Na signal contribution due to short-relaxation components was still appreciably high at TE = 2 ms (~46%), we elected to include these images in our estimation of long T_2_* component to improve the reliability of fitting (details below).

This was performed using both a log-linear and mono-exponential fit for comparison – see displays c) and d), yielding comparable relaxation times inside the left kidney of 24.7 and 23.8 ms, respectively. Log-linear fitting was chosen as the method for long-component T_2_* estimation on the healthy volunteer population due to increased speed of calculation. Log-linear fitting across a representative selection of voxels within regions of interest inside the abdomen was estimated to be ~36 times quicker to perform over an entire image volume than using mono-exponential fitting techniques.
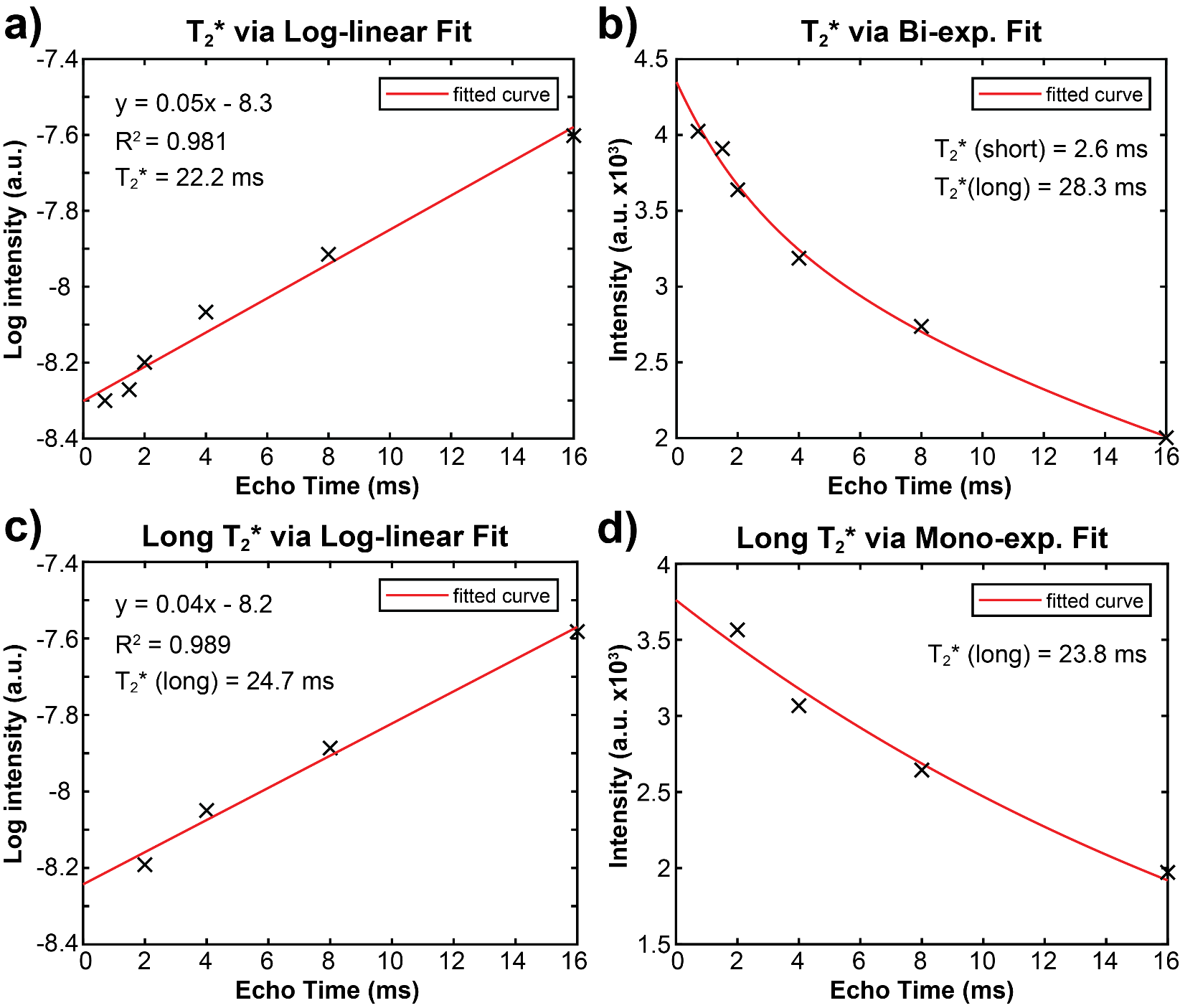


**Fig. S4** a) Example log-linear plot of ^23^Na signal intensity vs. pulse sequence echo time from a single axial slice region inside the left kidney of a healthy female volunteer (age 35-40). The ^23^Na T_2_* value was calculated by taking the inverse of the linear fit gradient through all six data points; b) bi-exponential curve fit of mean signal intensities across the same region in each echo time series image as defined in Equation S4 to estimate short and long T_2_* components; c, d) Estimation of the corresponding long T_2_* relaxation component via log-linear and mono-exponential fitting considering only the last four echo time series

# Evaluation of Population Average B_1_ Mapping

As outlined in the main text, a population average B_1_ map was created by taking the median non-zero value of fractional B_1_ intensity at each voxel across all healthy volunteers where DAM B_1_ maps were collected (N = 17). An example axial slice was shown in Figure 3f of the main text, but central slices through all projections are presented in displays a-c) of Figure S5 below for completeness. To evaluate the reliability of this method, the standard deviation of all non-zero fractional B_1_ intensities per voxel was calculated to determine the magnitude of B_1_ variation between subjects. A 3D median filter (kernel size 5 x 5 x 5, see Supporting Information Section 1) was applied after calculation of the standard deviation map for consistency with analysis methods presented elsewhere in the paper. These observations are illustrated in displays d-f) of Figure S5 below through central slices in each of the three imaging planes.


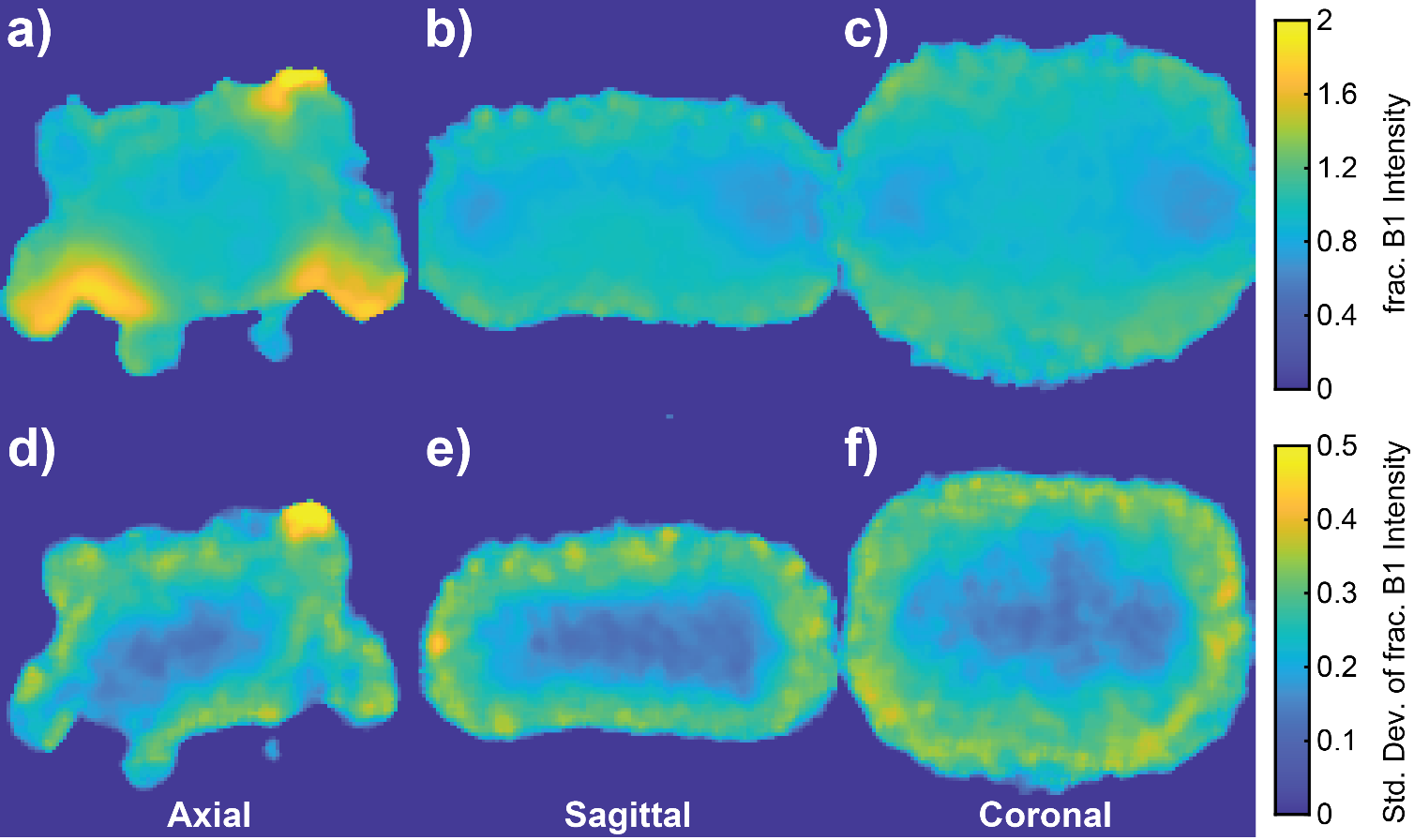


**Fig. S5** 3D median filtered maps of: a-c) median fractional B_1_ value and; d-f) fractional B_1_ standard deviation value across the healthy volunteer population where DAM B_1_ images were acquired. Maps are presented through central slices in each imaging plane.

As is visible from displays d-f) of Figure S5, the standard deviation in fractional B_1_ intensity across the healthy volunteer population was relatively small (~10-20%) in the central region of the coil where the majority of our organ regions of interest were located. To this end, we deemed that estimation of corrected TSC using our population average B_1_ method was reliable in this instance. Standard deviation was on average higher in voxels in proximity to the rungs of the body coil (as discussed in the main text), but also at the edges of the body at ~30-35%. This effect likely arises due to differences in subject body habitus, as voxels closer to the rungs of the coil were only non-zero (and thus included in median and standard deviation calculations) in a subset of the healthy volunteer population, making calculation of the standard deviation based on fewer points.

The region of high standard deviation visible in proximity to the location of the left 80 mM NaCl agar gel phantom in the top-right of display d) correlates to atypical phantom positioning in a single subject with a larger body habitus: fractional B_1_ in measured phantom region = 1.843. Removing this volunteer from the population average B_1_ map reduced the mean of standard deviation in fractional B_1_ values within the phantom region from 0.302 to 0.209. However, a recalculation of TSC with a population average B_1_ map excluding this volunteer was not deemed necessary to perform, since for the two subjects in question, a visual inspection of the high-resolution ^23^Na images indicated that the phantoms were not in close proximity to the rungs of the coil, and thus not in a region of high B_1_ variability.

# Total Sodium Concentration Measurements

All B_1_-corrected TSC values recorded from each organ in the healthy volunteer population are presented in Table S3 below for completeness, along with accompanying signal-to-noise ratio (SNR) measurements from the uncorrected signal intensity values from the high-resolution ^23^Na-MRI images. SNR was calculated by subtracting the mean signal intensity in a noise region from the mean signal intensity in the tissue regions of interest and dividing the result by the standard deviation of intensity values in the noise region.

| **IVC** | SNR | 25 | 25 | 23 | 25 | 24 | 19 | N/A | 19 | 22 | 21 | 28 | 24 | 19 | 26 | 25 | 18 | 30 | 23 | 14 | 28 | 22 | 4 |
| --- | --- | --- | --- | --- | --- | --- | --- | --- | --- | --- | --- | --- | --- | --- | --- | --- | --- | --- | --- | --- | --- | --- | --- |
|  | TSC | 83 | 62 | 84 | 108 | 78 | 58 | N/A | 45 | 108 | 95 | 92 | 78 | 126 | 95 | 55 | 87 | 78 | 88 | 51 | 100 | 83 | 21 |
| **Aorta** | SNR | 23 | 27 | 23 | 22 | 25 | 20 | N/A | 24 | 24 | 19 | 28 | 24 | 22 | 25 | 24 | 17 | 28 | 24 | 18 | 22 | 21 | 3 |
|  | TSC | 80 | 64 | 84 | 95 | 91 | 59 | N/A | 60 | 100 | 79 | 91 | 65 | 108 | 91 | 47 | 82 | 73 | 79 | 57 | 71 | 78 | 16 |
| **Spleen** | SNR | 12 | 16 | 17 | 15 | 16 | 14 | N/A | 14 | 12 | 13 | 15 | 16 | 12 | 15 | 38 | 10 | 16 | 14 | 10 | 15 | 13 | 3 |
|  | TSC | 37 | 30 | 43 | 68 | 35 | 39 | N/A | 29 | 35 | 52 | 42 | 34 | 40 | 35 | 32 | 44 | 37 | 42 | 32 | 47 | 40 | 9 |
| **Gall**  **Bladder** | SNR | 23 | 53 | 30 | 26 | 32 | 57 | N/A | 19 | 16 | 16 | 32 | 53 | 28 | 40 | 23 | N/A | 55 | 18 | 33 | 63 | 33 | 15 |
|  | TSC | 97 | 140 | 108 | 123 | 129 | 197 | N/A | 56 | 85 | 74 | 103 | 203 | 155 | 122 | 79 | N/A | 138 | 80 | 122 | 193 | 122 | 42 |
| **Liver** | SNR | 11 | 13 | 14 | 14 | 14 | 11 | N/A | 13 | 12 | 10 | 14 | 14 | 12 | 15 | 23 | 10 | 16 | 12 | 8 | 14 | 11 | 3 |
|  | TSC | 41 | 33 | 46 | 46 | 42 | 32 | N/A | 28 | 44 | 42 | 39 | 35 | 43 | 42 | 31 | 73 | 37 | 47 | 29 | 45 | 41 | 10 |
| **CSF** | SNR | 34 | 33 | 29 | 30 | 37 | 28 | N/A | 30 | 29 | 28 | 36 | 33 | 32 | 29 | 42 | 22 | 35 | 32 | 26 | 33 | 30 | 4 |
|  | TSC | 105 | 70 | 99 | 106 | 118 | 79 | N/A | 70 | 110 | 113 | 104 | 87 | 131 | 84 | 68 | 74 | 93 | 90 | 72 | 105 | 94 | 18 |
| **Right**  **Kidney** | SNR | 28 | 30 | 27 | 24 | 30 | 26 | N/A | 28 | 25 | 22 | 28 | 27 | 26 | 28 | 45 | 21 | 29 | 26 | 20 | 31 | 26 | 5 |
|  | TSC | 69 | 70 | 76 | 77 | 85 | 70 | N/A | 59 | 76 | 74 | 78 | 64 | 80 | 73 | 59 | 68 | 64 | 68 | 55 | 91 | 71 | 9 |
| **Left**  **Kidney** | SNR | 30 | 31 | 27 | 21 | 28 | 20 | N/A | 24 | 23 | 21 | 26 | 25 | 20 | 22 | 40 | 18 | 26 | 26 | 20 | 24 | 23 | 5 |
|  | TSC | 110 | 57 | 88 | 83 | 97 | 61 | N/A | 63 | 82 | 97 | 74 | 61 | 104 | 61 | 78 | 67 | 68 | 89 | 70 | 88 | 79 | 15 |
| **Vol.** | | **1** | **2** | **3*** | **4** | **5** | **6*** | **7** | **8** | **9** | **10** | **11** | **12** | **13** | **14** | **15** | **16** | **17** | **18** | **19** | **20** | **Mean** | **St.Dev** |

**Table S3** Mean B_1_-corrected TSC values and mean uncorrected high-resolution ^23^Na-MRI image signal-to-noise ratio (SNR) values calculated in each abdominal organ or structure for the 19 healthy volunteers. Volunteers marked with an asterisk indicate that no DAM B_1_ mapping was performed as part of the protocol, and that the TSC calculations for these subjects instead utilised the population average estimate for B_1_

Mean values across the healthy volunteer population are presented in Figure 5 of the main text, along with associated standard deviation error bars. No TSC values are quoted for Volunteer 7 where the high-resolution ^23^Na series was not acquired, or for the gall bladder in Volunteer 16 due to previous organ removal.

# Long-Component ^23^Na T_2_* Measurements

All long-component T_2_* values recorded from each organ in the healthy volunteer population are presented in Table S4 below for completeness. Mean values across the healthy volunteer population are presented in Figure 7 of the main text, along with associated standard deviation error bars. No long-component T_2_* values are quoted for Volunteer 2 where the low-resolution ^23^Na variable-echo time series were not acquired, or for the gall bladder in Volunteer 16 due to previous organ removal.

| **Volunteer** | **Long-Component T_2_* Relaxation Time (ms)** | | | | | | | | |
| --- | --- | --- | --- | --- | --- | --- | --- | --- | --- |
|  | **Phantom** | **Left kidney** | **Right kidney** | **CSF** | **Liver** | **Gall Bladder** | **Spleen** | **Aorta** | **IVC** |
| **1** | 6.2 | 26.8 | 28.8 | 29.8 | 10.2 | 12.3 | 14.6 | 17.5 | 18.3 |
| **2** | N/A | N/A | N/A | N/A | N/A | N/A | N/A | N/A | N/A |
| **3** | 5.5 | 16.6 | 19.5 | 15.3 | 11.0 | 9.7 | 4.3 | 17.2 | 13.3 |
| **4** | 8.9 | 21.9 | 21.8 | 29.0 | 11.6 | 13.0 | 14.8 | 21.1 | 16.0 |
| **5** | 12.3 | 23.2 | 20.4 | 27.0 | 17.4 | 9.8 | 17.8 | 22.3 | 18.2 |
| **6** | 6.9 | 21.4 | 19.0 | 24.5 | 14.7 | 12.6 | 6.8 | 14.6 | 14.9 |
| **7** | 11.7 | 25.4 | 26.0 | 20.8 | 8.6 | 15.1 | 13.1 | 21.8 | 18.3 |
| **8** | 6.5 | 21.7 | 17.5 | 24.3 | 8.4 | 15.4 | 7.9 | 18.2 | 14.1 |
| **9** | 8.2 | 25.9 | 24.2 | 24.3 | 11.9 | 12.0 | 10.2 | 17.8 | 13.3 |
| **10** | 5.4 | 24.2 | 25.9 | 23.5 | 11.4 | 15.4 | 11.9 | 23.0 | 23.8 |
| **11** | 14.7 | 20.5 | 18.5 | 28.4 | 8.0 | 15.7 | 5.5 | 12.8 | 9.6 |
| **12** | 6.6 | 23.9 | 22.8 | 23.0 | 11.0 | 29.9 | 8.5 | 6.1 | 6.3 |
| **13** | 5.9 | 21.6 | 23.0 | 33.3 | 7.3 | 15.0 | 5.7 | 13.3 | 12.5 |
| **14** | 6.8 | 24.0 | 24.9 | 25.5 | 13.2 | 20.6 | 13.6 | 19.3 | 19.3 |
| **15** | 11.1 | 24.7 | 20.7 | 19.3 | 6.9 | 10.4 | 5.1 | 15.8 | 14.3 |
| **16** | 7.7 | 24.8 | 22.2 | 17.0 | 9.1 | N/A | 17.4 | 14.3 | 18.3 |
| **17** | 10.1 | 21.0 | 24.4 | 22.5 | 9.7 | 24.0 | 7.0 | 15.8 | 16.5 |
| **18** | 6.8 | 25.6 | 25.3 | 27.0 | 10.5 | 11.1 | 10.0 | 21.5 | 19.0 |
| **19** | 6.1 | 25.5 | 24.7 | 29.3 | 12.4 | 26.9 | 11.0 | 19.0 | 17.3 |
| **20** | 6.9 | 20.3 | 24.4 | 22.0 | 13.3 | 25.4 | 12.4 | 20.8 | 20.0 |
| **Mean** | 8.2 | 23.2 | 22.7 | 24.6 | 10.7 | 15.8 | 10.3 | 17.3 | 15.7 |
| **St.Dev.** | 2.7 | 2.5 | 3.1 | 4.6 | 2.7 | 6.0 | 4.2 | 4.2 | 4.0 |

**Table S4** Mean T_2_* values calculated in one 80 mM agar phantom (patient left chosen in all cases for consistency) and each abdominal organ or structure for the 19 healthy volunteers

# References Used in Supporting Information

1. Romanzetti S, Mirkes CC, Fiege DP, Celik A, Felder J, Shah NJ (2014) Mapping tissue sodium concentration in the human brain: A comparison of MR sequences at 9.4Tesla. NeuroImage 96:44-53.

2. Block KT, Frahm J (2005) Spiral imaging: a critical appraisal. J Magn Reson Imaging 21 (6):657-668.

3. Zeng H, Constable RT (2002) Image distortion correction in EPI: comparison of field mapping with point spread function mapping. Magn Reson Med 48 (1):137-146.

4. Zbyn S, Juras V, Michaely HJ, Deligianni X, Bieri O, Schoenberg SO, Trattnig S, Haneder S (2013) Sodium T2* Mapping of the Human Kidneys in Vivo at 7 Tesla. Proc Intl Soc Mag Reson Med 21:4142.

5. Maril N, Margalit R, Mispelter J, Degani H (2004) Functional sodium magnetic resonance imaging of the intact rat kidney. Kidney International 65 (3):927-935.

6. Sharafi A, Chang G, Regatte RR (2018) Biexponential T(2) relaxation estimation of human knee cartilage in vivo at 3T. J Magn Reson Imaging 47 (3):809-819.

7. Gomolka RS, Ciritsis A, Meier A, Rossi C (2020) Quantification of sodium T1 in abdominal tissues at 3 T. Magnetic Resonance Materials in Physics, Biology and Medicine 33 (3):439-446.

8. Bansal N, Germann MJ, Seshan V, Shires GT, 3rd, Malloy CR, Sherry AD (1993) Thulium 1,4,7,10-tetraazacyclododecane-1,4,7,10-tetrakis(methylene phosphonate) as a 23Na shift reagent for the in vivo rat liver. Biochemistry 32 (21):5638-5643.

9. Madelin G, Regatte RR (2013) Biomedical applications of sodium MRI in vivo. J Magn Reson Imaging 38 (3):511-529.
